# Small extrachromosomal circular DNA harboring targeted tumor suppressor gene mutations supports intratumor heterogeneity in mouse liver cancer induced by multiplexed CRISPR/Cas9

**DOI:** 10.1186/s13073-023-01230-2

**Published:** 2023-10-06

**Authors:** Tao Guo, Guo-Qiao Chen, Xu-Fan Li, Meng Wang, Kun-Ming Liu, Xiao-Ying Yang, Si-Cheng Liu, Yi-Li Feng, Peng-Yuan Liu, Hui Lin, An-Yong Xie

**Affiliations:** 1 Innovation Center for Minimally Invasive Technique and Device, Department of General Surgery, Sir Run Run Shaw Hospital, Zhejiang University School of Medicine, 3 East Qingchun Rd, Hangzhou, Zhejiang 310019 P. R. China; 2 Institute of Translational Medicine, Zhejiang University School of Medicine and Zhejiang University Cancer Center, 268 Kai Xuan Rd, Hangzhou, Zhejiang 310029 P. R. China

**Keywords:** CRISPR, Cas9, Somatic multiplex-mutagenesis, Liver tumor, Intratumor genetic heterogeneity, eccDNA

## Abstract

**Background:**

Primary liver cancer has significant intratumor genetic heterogeneity (IGH), which drives cancer evolution and prevents effective cancer treatment. CRISPR/Cas9-induced mouse liver cancer models can be used to elucidate how IGH is developed. However, as CRISPR/Cas9 could induce chromothripsis and extrachromosomal DNA in cells in addition to targeted mutations, we wondered whether this effect contributes to the development of IGH in CRISPR/Cas9-induced mouse liver cancer.

**Methods:**

CRISPR/Cas9-based targeted somatic multiplex-mutagenesis was used to target 34 tumor suppressor genes (TSGs) for induction of primary liver tumors in mice. Target site mutations in tumor cells were analyzed and compared between single-cell clones and their subclones, between different time points of cell proliferation, and between parental clones and single-cell clones derived from mouse subcutaneous allografts. Genomic instability and generation of extrachromosomal circular DNA (eccDNA) was explored as a potential mechanism underlying the oscillation of target site mutations in these liver tumor cells.

**Results:**

After efficiently inducing autochthonous liver tumors in mice within 30–60 days, analyses of CRISPR/Cas9-induced tumors and single-cell clones derived from tumor nodules revealed multiplexed and heterogeneous mutations at target sites. Many target sites frequently displayed more than two types of allelic variations with varying frequencies in single-cell clones, indicating increased copy number of these target sites. The types and frequencies of targeted TSG mutations continued to change at some target sites between single-cell clones and their subclones. Even the proliferation of a subclone in cell culture and in mouse subcutaneous graft altered the types and frequencies of targeted TSG mutations in the absence of continuing CRISPR/Cas9 genome editing, indicating a new source outside primary chromosomes for the development of IGH in these liver tumors. Karyotyping of tumor cells revealed genomic instability in these cells manifested by high levels of micronuclei and chromosomal aberrations including chromosomal fragments and chromosomal breaks. Sequencing analysis further demonstrated the generation of eccDNA harboring targeted TSG mutations in these tumor cells.

**Conclusions:**

Small eccDNAs carrying TSG mutations may serve as an important source supporting intratumor heterogeneity and tumor evolution in mouse liver cancer induced by multiplexed CRISPR/Cas9.

**Supplementary Information:**

The online version contains supplementary material available at 10.1186/s13073-023-01230-2.

## Background

Cancer is driven by genetic changes, which include point mutations, small insertions and deletions (indel), large genome rearrangements, and copy number alterations (CNAs) [1]. These genetic alterations in a single genome manifest genomic instability, a hallmark of cancer, and are generated directly or indirectly by repair of DNA damage induced by exogenous agents or from endogenous sources. This mutational process continues to operate throughout tumor evolution and may lead to phenotypic variations between cells within the final neoplasm. The genetic and phenotypic heterogeneity within a tumor nodule, defined as intratumor heterogeneity (ITH), fuels tumor growth that would follow the fundamental principles of Darwinian evolution [2, 3]. In this model, genetic mutations are continuously generated and gradually selected for differential fitness of daughter cells in tumor growth. However, growing evidence indicates that diverse genetic alterations may occur early in bursts in many types of cancers and their pervasive distributions in tumors often fit neutral growth pattern [2, 4]. Catastrophic events such as chromothripsis could also initiate transformation of tumor cells and result in ITH early in one or a few cell divisions of tumor cells [4–8]. Thus, punctuated evolution or the “Big Bang” theory associated with these mutational processes has been proposed to explain the observation of more extensive ITH than that predicted by positive selection in many types of cancers [8–14].

Molecular analysis of primary liver cancer revealed significant ITH, which is attributable to the difficulty in effective treatment of liver cancer [15]. Primary liver cancer ranks the seventh in term of cancer incidence and the third in cancer-related mortality worldwide. Hepatocellular carcinoma (HCC) is the most common form of liver cancer and accounts for ~ 90% of cases [16]. Despite improved understanding of liver cancer, the ITH development remains one of the fundamental questions in biology of this disease. Mouse models provide an important tool to study the ITH of liver cancer [17]. CRISPR/Cas9, a powerful gene editing technology, has been used to rapidly generate mouse models for human cancer by inactivating tumor suppressor genes (TSGs) or activating oncogenes in somatic cells [18]. CRISPR/Cas9-mediated targeted inactivation of *p53* and *Pten* in mouse liver induced intrahepatic cholangiocarcinoma (ICC), a second form of primary liver cancer, in mice [19]. Multiplexing CRISPR/Cas9 has also been explored for high-throughput analysis of TSGs for initiation and development of liver cancer [20–24]. In these applications, CRISPR/Cas9 induces DNA double-strand breaks (DSBs) in somatic cells and repair of these DSBs by either non-homologous end joining (NHEJ) or homology-directed repair (HDR) generates edited products that induce neoplastic transformation [25].

Recent studies indicate that CRISPR/Cas9-induced DSBs could also lead to chromothripsis and generation of extrachromosomal circular DNA (eccDNA) in cells, in addition to targeted mutations [26, 27]. EccDNA can be inherited and integrated back into the genome of tumor cells during tumor growth, thus expanding intratumor genetic heterogeneity (IGH) [28–32]. It was recently reported that oncogenes on eccDNA elements (eccDNAs) with copy number oscillation drive tumor evolution and resistance to cancer therapy, although the contributions of eccDNAs remains difficult to identify in the cancer genome due to relatively low incidence and technological barriers [29, 33–35]. Still, several sequencing methods have been developed for genome-wide identification and mapping of small eccDNAs (also called microDNA) that range in size from 0.1 kb to 2 kb [36–40]. It has been shown that these small eccDNAs are generated primarily from exons, 5′-untranslated regions (UTRs) and 3′-UTRs [36, 41, 42]. In CRISPR/Cas9-based development of mouse models for primary liver cancer, simultaneous induction of numerous DSBs by multiplexing CRISPR/Cas9 may amplify genomic instability that is potentially marked with eccDNA, adding a layer of genetic variations into ITH of mouse liver cancer [26, 27]. Additionally, many targeted mutations of TSGs in the founder tumor cells may not be a driver, but they could support the development of ITH and participate tumor evolution in the later stage of tumor development. It is however unclear how these targeted mutations, a driver or not, engage the ITH development. Here, by simultaneously targeting 34 TSGs, we rapidly generated CRISPR/Cas9-based liver cancer in mice. By tracking targeted mutations during clonal expansion of single primary liver tumor cells derived from mouse liver tumor nodules, we discovered that the copy number of targeted mutations oscillated over time, even between null and not null, in vitro and in vivo. We found that these cells were associated with significant genomic instability and contained small eccDNAs harboring targeted TSG mutations. This suggests that small eccDNAs carrying TSG mutations may support the development of ITH in primary liver cancer.

## Methods

### Cell culture and transfection

Murine fibroblast cell line NIH-3T3, hepatoma cell line Hepa1-6, and primary murine liver cancer cells were grown in Dulbecco’s Modified Eagle’s Medium supplemented with 10% fetal bovine serum (FBS) (Gibco), 100 IU/ml penicillin (Gibco), and 100 μg/ml streptomycin (Gibco) at 37 °C with 5% CO_2_. Transfection in cell line was performed with Lipofectamine 2000 transfection reagent (Invitrogen) in 24-well plates according to the manufacturer’s protocol.

### Target TSG selection and sgRNA design and cloning

Thirty-four target TSGs were selected according to the following criteria: (1) TSGs frequently mutated in primary human liver cancer and (2) TSGs recently reported as candidate tumor suppressor genes in primary human liver cancer. Selected TSGs were also involved in at least 11 cancer-related signaling pathways. The 5′-upstream untranslated site of the *Setd5* locus close to the safe-harbor *Rosa26* locus was chosen as a genome editing control. The *Streptococcus pyogenes* Cas9 (*Sp*Cas9)-sgRNA plasmid px330 was reconstructed to generate separate expression plasmids for *Sp*Cas9 and sgRNA. Single *p53* sgRNA sequence described previously was chosen and constructed into the sgRNA expression plasmid [19]. Single *Setd5* sgRNA sequence was also chosen as the genome editing control. For the remaining 33 TSGs, 3 sgRNAs each gene were designed and constructed as described previously [43]. All constructs were validated by Sanger sequencing.

### Identification of suitable sgRNAs for somatic mutagenesis of 34 TSGs

To identify a single efficient sgRNAs from 3 sgRNA candidates for each of 34 TSGs, we modified the BGN reporter previously established to determine sgRNA efficiency [43]. Briefly, 23-nt oligodeoxynucleotides (ODNs) for sgRNA targets were ordered, annealed, and inserted into the I-SceI-EcoRI site within the linker region of the *Blasticidin S deaminase* (*BsdR*)*-GFP* fusion in the BGN reporter. As a mutagenic NHEJ reporter, the modified *GFP*-based BGN reporter transfected into cells produced out-of-frame GFP after transcription and translation and thereby no GFP^+^ cells [43]. A site-specific DSB was induced at the BGN reporter in NIH-3T3 cells after co-transfection of the expression plasmids for *Sp*Cas9 and sgRNAs with the modified BGN reporter containing the *Sp*Cas9-sgRNA target. Mutagenic NHEJ of this DSB could generate indels inducing “3n + 1”-bp frame-shift, thus reframing GFP to the correct frame and making the cell GFP^+^. GFP^+^ cells could be measured by flow cytometry. Therefore, the percentages of GFP^+^ cells at 72 h post-transfection could reflect the efficiency of sgRNA-guided *Sp*Cas9 editing, and effective sgRNAs be identified.

### Animal experiments and animal work statement

Six-week male wide-type C57BL/6 mice and severe combined immunodeficient (SCID) mice were purchased from Shanghai SLAC Laboratory Animal Co. and were maintained in a specific pathogen-free (SPF) room under a 12-h light/12-h dark cycle at the Animal Research Center of the Institute of Translation Medicine School of Medicine, Zhejiang University. To induce primary mouse liver cancer, a total of 200 μg *Sp*Cas9 plasmid and 35 μg of sgRNAs were mixed in 0.9% NaCl solution with 1.5–2.0 mL volume and the mixture that accounts for 8–10% of body weight in amount was injected into C57BL/6 mice via tail vein within 10 s. For dosage experiments, each sgRNA was used at 0.05–1.0 μg, and the sgRNA empty vector was added to 35 μg in total for the sgRNA expression plasmids. At day 30, 90, and 120 after injection, mice were sacrificed and examined for tumor formation. Mouse livers and liver tumors were collected to make formalin-fixed paraffin-embedded (FFPE) tissues or to be fixed with liquid nitrogen for gDNA extraction. For subcutaneous tumor grafts, 1 × 10^6^ 1C3-1 cells were suspended in 200 µL phosphate-buffered saline (PBS) and inoculated subcutaneously at inguinal region of SCID mice. The tumor formation was examined daily and the tumors harvested at day 14 post inoculation. Mice in all experiments were euthanized with carbon dioxide before being sacrificed for tumor analysis. All animal experiments were carried out according to the animal ethics guidelines and approved protocols from the Animal Care and Use Committee of Zhejiang University, with the approval number ZJU2015– 378–01.

### Mouse liver tissue dissection, histology, and immunohistochemistry

Five-micrometer slices of non-tumor and tumor FFPE tissues were stained with hematoxylin and eosin (HE) and analyzed under a microscope (Leica DM4000, Germany). Tumor tissues were evaluated for expression of alpha-fetoprotein (AFP), cytokeratin 19 (CK19), and Golgi glycoprotein 73 (GP73) by immunohistochemistry. Briefly, 5 μm FFPE tumor sections were blocked with goat serum for 30 min, incubated with mouse anti-AFP (1:50), anti-CK19 (1:50), or anti-GP73 (1:100) monoclonal antibodies (Santa Cruz Biotechnology, USA) at 4 °C overnight and further stained with 3,3′-diaminobenzidine at room temperature for 3 min. Expression of AFP, CK19, and GP73 were examined by microscope.

### Establishment of primary mouse liver cancer cell lines and single-cell clones

Fresh mouse primary liver tumor tissues were obtained within 1 h after mice were sacrificed under sterile conditions. The tissues were minced into less than 1 mm^3^ small pieces using scissors, washed with PBS and centrifuged at 1000 rpm for 5 min. The tissue precipitates were digested with 0.1% trypsin at 37 °C for 30 min before DMEM medium containing 10% FBS was added to stop digestion. The suspension was filtered by 400-mesh cell sieve and centrifuged at 1000 rpm for 5 min. Cell pellets were resuspended in complete culture medium and plated into a 10-cm dish to establish primary mouse liver cancer cell lines. To establish single-cell clones, 100 single cells were diluted into 8-mL culture medium and plated into a 10-cm dish. After 10–14 days, single clone was visible, picked under a microscope using plastic clonal ring and transferred into a 96-well plate for cell expansion.

### Immunofluorescence for liver cancer biomarkers and DNA damage response analysis

To analyze spontaneous γH2AX and 53BP1 focus formation in primary liver cancer cells, cells were fixed in 4% paraformaldehyde for 10 min on ice, permeabilized with 0.5% Triton 100 for 10 min on ice, and blocked with 1% bovine serum albumin for 30 min. Subsequently, cells were stained with primary antibodies mouse anti-γH2AX monoclonal antibody (Abcam) or rabbit anti-53BP1 polyclonal antibody (Abcam) overnight and then with secondary antibody for 2 h. Cell nuclei were stained by 4′,6-diamidino-2-phenylindole (DAPI). γH2AX and 53BP1 foci were determined and counted under fluorescence microscope. Micronuclei were also stained with DAPI and counted. All experiments were repeated at least three times and images were captured by microscope.

### Detection of stable Cas9 activity in cancer cell lines

Western blotting was performed to detect *Sp*Cas9 protein in primary liver tumor cell lines derived from CRISPR/Cas9-induced mouse liver tumors. Cells were washed with cold PBS for two times, and then lysed in Radio-Immunoprecipitation Assay buffer containing 1 × protease inhibitor and 1 mM PMSF on ice for 15 min. After centrifugation of lysis extracts at 4 °C, 10 μg proteins from suspension were separated on 10% sodium dodecyl sulfate polyacrylamide gel and transferred onto polyvinylidene fluoride membranes. Membranes were blocked with non-fat milk at room temperature for 1 h, washed with TBST buffer, and incubated with mouse anti-FLAG antibody (HUABIO, China) at 4 °C overnight with anti-GAPDH as loading control. Protein bands for *Sp*Cas9 and GAPDH were captured by Bio-Rad ChemiDoc Touch. To determine stable *Sp*Cas9 activity in primary liver tumor cell lines, we transfected these cells with the positive sgRNA control and performed targeted PCR amplicon and deep sequencing to evaluate editing efficiency on the targeted sites. Experiments were repeated three times in cell line 1C3-1 and done once in other cell lines.

### Metaphase spread analysis of ploidy and chromosomal aberrations

Cells were plated in 10 cm dish, grown to 70% confluence and treated with Colchicine in culture at 1.0 μg/mL for primary liver cancer cell line, 0.8 μg/mL for NIH-3T3 and Hepa1-6 cells and cells for 6 h. Cells were then harvested with 0.25% trypsin, resuspended in 75 mM KCl solution at 37 °C for 20–25 min and centrifuged at 1500 rpm for 5 min. After being fixed with methanol-acetic acid (3:1) for 15 min twice, the cells were dripped onto cold glass slides from 30 cm height and stained with 5% Giemsa for 10 min. Chromosome number and chromosomal aberration were assessed under light microscopy (× 100). Images were analyzed by Image J.

### Purification of genomic DNA and targeted PCR amplicon deep sequencing

Genomic DNA (gDNA) was extracted from cells and 20 mg fresh liver tumor tissues using the Genomic DNA Miniprep Kit (AxyGEN, USA) according to the manufacturer’s instructions. As done previously [44], 35 targeted sites of gDNA and eccDNAs were amplified by PCR with primers listed (Additional file 1: Table S1). PCR products were purified by AxyPrep DNA Gel Extraction Kit or AxyPrep PCR Cleanup Kit (AxyGEN). Purified PCR products of each target were combined into a tube with equal volume. One microgram of PCR product mixture was used to establish DNA fragment libraries by VAHTS Universal DNA Library Prep Kit for Illumina V2 (Vazyme Biotech, China) and sequenced on Illumina HiSeq 2500.

### Illumina sequencing data analysis, variant calling, and clustering of variant frequencies

Sequencing reads were aligned and processed according to Illumina sequencing analysis procedures and further analyzed by our analysis software developed previously [43, 44]. Variant metrics including sequences and read depths was generated using in-house python scripts. Mutation frequency for each target gene in tissues and cells was calculated as total edited read count of the gene divided by total read count of the gene. To calculate allelic mutation ratio, the depth of sequence covering each mutation was divided by the overall read count of the corresponding gene. For each sample, we normalized read count of a specific gene by the library size to generate read ratios for gDNA and eccDNA separately. Data visualization was performed in R (v3.6.2) by ggplot2 package.

### Purification of eccDNA and Circle-Seq

Genome-wide characterization of eccDNA was performed using Circle-Seq after eccDNA purification as described previously [45]. The eccDNA purification and Circle-Seq protocol consisted of multiple steps including cell lysis, eccDNA enrichment by column chromatography, removal of mitochondrial DNA by the restriction endonuclease PacI and linear DNA by Plasmid-Safe ATP-Dependent DNase, rolling circle amplification (RCA) of eccDNA, library preparation, and deep sequencing. In brief, a total of 1 × 10^7^ cells from the control cells NIH3T3, the clone 1C3-1, and 6C7 were collected and suspended in Buffer P1 (Vazyme, Catalog: #DC203-01) and lysed using 15 μL Proteinase K (YEASEN, Catalog: #10412ES03) at 50 °C for 48 h. Crude eccDNAs were enriched using an ion-exchange column with the FastPure EndoFree Plasmid Mini Kit (Vazyme, Catalog: #DC203-01). Mitochondrial DNA was removed using PacI (New England Biolabs, Catalog: #R0547V), and linear DNA was further eliminated using 15 U of Plasmid-Safe ATP-Dependent DNase (Epicentre, Catalog: #E3110K) per day at 37 °C for 10 days. The purified eccDNAs were then amplified using Phi29 DNA polymerase (Thermo Fisher, Catalog: #EP0091) at 30 °C for 48 h. Libraries were prepared from ~ 200 ng purified fragmented DNA. After library preparation, deep sequencing was performed on an Illumina NovaSeq 6000 sequencer using Illumina paired-end mode. Sequence reads were mapped to a mouse reference genome to record the origin of chromosome-derived small eccDNAs by Circle-MAP software [46].

### PCR validation in removal of mitochondrial DNA and linear DNA from eccDNA

To validate removal of mitochondrial DNA and linear DNA, 50 ng each of crude eccDNA and eccDNA purified, with the same amount of gDNA as a positive control, was used to amplify targeted regions of the mouse gene *Actb* and *Cox5b* and the mouse mitochondrial gene *mt-Co1* [41, 45]. PCR products were resolved by DNA gel electrophoresis and stained by either ethidium bromide or SYBR Gold stain (Thermo Fisher, Catalog: #S11494) for higher sensitivity [47]. The primer pairs used for PCR included the following: 5′-GAGACCTTCAACACCCCAG-3′ (forward) and 5′-TCAGGGCATCGGAACCG-3′ (reverse) for a 404-bp region of *Actb*, 5′-GCCCATTTCCACTATGTTCTA-3′ (forward) and 5′-AGTAGCCTGCTCCTCATCAG-3′ (reverse) for a 119-bp region of *Cox5b*, and 5′-GCCCATTTCCACTATGTTCTA-3′ (forward) and 5′-GTTTACTCCTACGAATATGATG-3′ (reverse) for a 144-bp region of *mt-Co1*.

### Sanger sequencing of eccDNA circularization junctions

To identify circularization junction of eccDNAs harboring a TSG target site or neighboring a TSG target site, outward PCR primers were designed and positioned on one side to the TSG target site (Additional file 1: Table S1). Outward PCR were performed using purified eccDNAs as a template. Formation of eccDNA was further validated by inward PCR of eccDNA with inward PCR primer pair listed in Additional file 1: Table S1. PCR products were verified first by DNA gel analysis. Visible PCR bands were purified and cloned into pUC19 vector and sequenced by Sanger sequencing. The sequences were analyzed to identify circularization junction of eccDNAs purified from 1C3-1cells.

## Results

### Induction of primary mouse liver cancer by multiplexed CRISPR/Cas9 targeting 34 TSGs

**Fig. 1:**
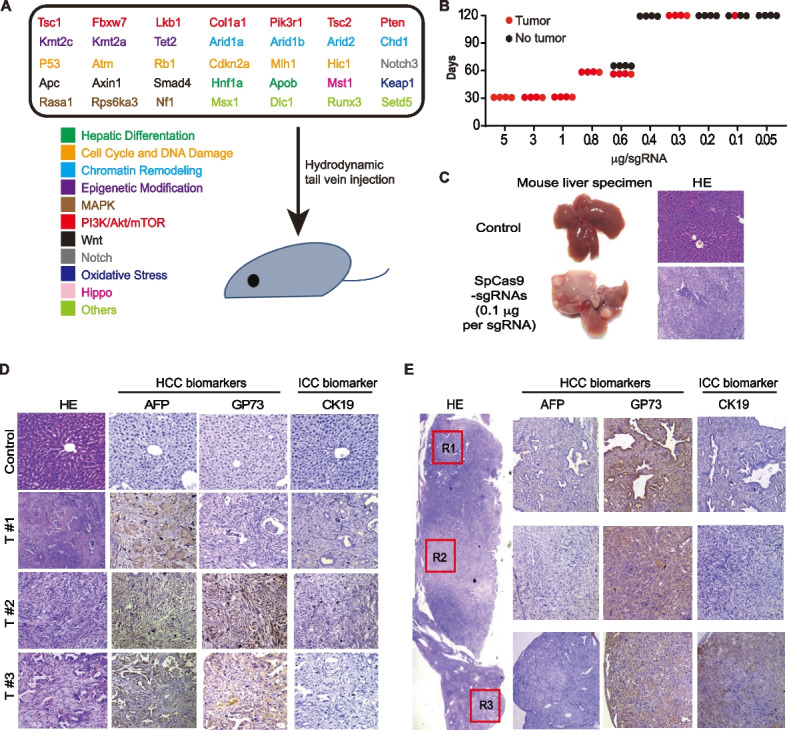
Induction of primary mouse liver cancer by CRISPR/Cas9-mediated somatic mutagenesis in mice. **A** 34 TSGs and the Setd5 control site targeted by multiplexed CRISPR/Cas9. Each TSG is indicated with its related cancer signaling pathway. Hepatic delivery of *Sp*Cas9 and sgRNA expression plasmids is achieved by hydrodynamic tail vein injection (HTVI). **B** Effect of sgRNA dosage on CRISPR/Cas9-induced liver tumor formation. Each dot indicates one mouse. **C** Mouse liver specimen with or without tumor nodules (left) and H&E staining of a tumor nodule (right). **D** Microscopic IHC images of CRISPR/Cas9-induced liver tumors. T#1, T#2, and T#3 represent tumor nodules from three mice. AFP and GP73: HCC biomarkers; CK19: ICC biomarker. **E** Histology and IHC staining of a liver tumor nodule indicating three heterogeneous regions. Left: these three regions indicated with R1, R2, and R3 in H&E staining; Right: microscopic IHC images for these three regions

To induce mouse liver tumors using multiplexed CRISPR/Cas9, we chose 34 TSGs along with a 5′-upstream site of the *Setd5* locus as a negative control for CRISPR/Cas9-mediated gene inactivation (Fig. 1A; Additional file 2: Fig. S1). These 34 TSGs are involved in at least 11 cancer-related signaling pathways (Fig. 1A). We designed and constructed at least 3 sgRNAs for each target gene except *p53* and analyzed these sgRNAs for their efficiency of frame-shift editing using our BGN reporter established previously (Additional file 2: Fig. S2A) [43]. The 23-bp target sequence containing the PAM for each sgRNA was inserted into the I-SceI-EcoRI site of the BGN reporter (Additional file 2: Fig. S2A). To test each sgRNA, the expression plasmids for Cas9 and sgRNA and the BGN reporter plasmid containing the Cas9-sgRNA target were transfected into mouse embryonic fibroblast cell line NIH-3T3 cells and the frequencies of “3n + 1”-bp frame-shift measured by flow cytometry for GFP^+^ cells (Additional file 2: Fig. S2B). After testing of each sgRNA, we selected the most effective sgRNA for each target gene to establish a plasmid library of 35 sgRNAs. We then used hydrodynamic tail vein injection (HTVI) to deliver this sgRNA plasmid library mixed with the *Streptococcus pyogenes* Cas9 (*Sp*Cas9) expression plasmid into mouse liver cells to induce liver tumors [19, 48]. Total volume injected was 2 mL with the amount of the *Sp*Cas9 plasmid fixed at 200 μg for each mouse. The amounts of each sgRNA in the sgRNA library injected ranged from 0.005 μg to 5 μg (Fig. 1B). Liver tumors were formed and visible in mice within 30–60 days (Fig. 1B–C). Dilution of sgRNA delayed development of Cas9/sgRNA-induced mouse liver cancer and reduced cancer induction (Fig. 1B). After each sgRNA was reduced to 0.2 μg, tumor development rarely occurred (Fig. 1B). Histological analysis of tumor nodules indicated that the hepatic lobule structures were destructed in tumor nodules (Fig. 1D). Immunostaining for the HCC biomarkers AFP and GP73 and the ICC biomarker CK19 revealed the presence of HCC, ICC, and mixed HCC-ICC types in mouse liver tumors induced by CRISPR/Cas9 (Fig. 1D). In one section of a single tumor nodule, three selected regions exhibited distinct histologic features (Fig. 1E), indicating strong ITH in CRISPR/Cas9-induced liver cancer in mice.

### Heterogeneity of targeted TSG mutations in CRISPR/Cas9-induced mouse liver tumors

**Fig. 2:**
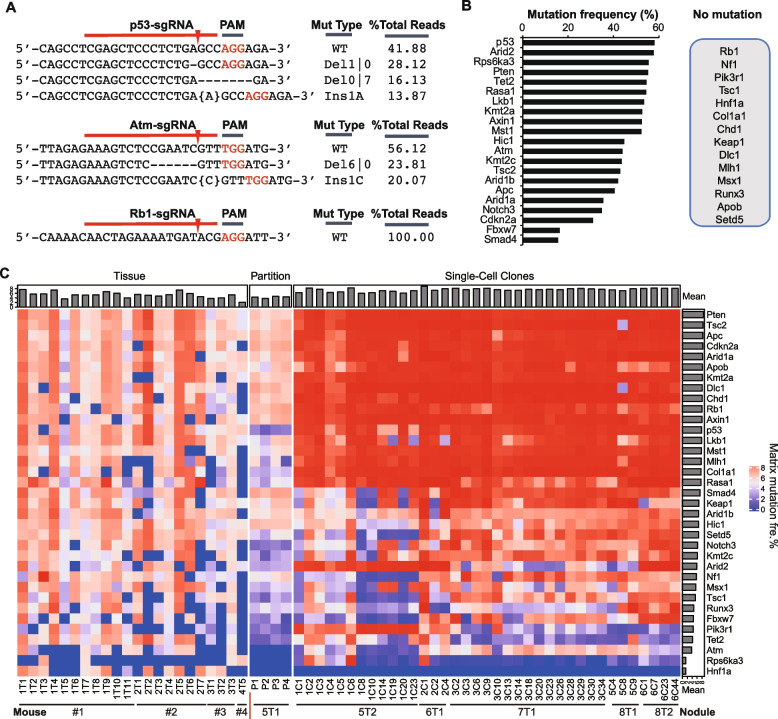
Target TSG mutation profiles in CRISPR/Cas9-induced mouse liver tumor nodules and single-cell clones. **A** Representative target mutation types of *p53*, *Atm* and *Rb1* in a tumor nodule induced by *Sp*Cas9 together with 35 sgRNAs each at 0.8 mg and the frequencies of these mutations. sgRNA target sites with PAM and the cleavage position are indicated. Dash lines and nucleotides in curly bracket in the sequences denote deletion and inserted nucleotides, respectively. WT, wild-type; Del, deletion; Ins, insertion. The numbers flanking Del or Ins indicate the numbers of deleted or inserted nucleotides. **B** Representative target mutations of 21 TSGs with cumulative frequencies in the tumor nodule in **A**. Fourteen unedited gene are also listed on the right. **C** Mutational landscape of 35 targets in 22 primary tumor nodules, 4 parts from a single tumor nodule, and 37 single-cell clones as indicated. Top and right bars indicate the mean of the mutation frequencies for each sample and each gene, respectively. Cumulative matrix mutation frequencies are highlighted from the highest 8 in red to the lowest 0 in blue

We performed next generation sequencing (NGS) of PCR-amplified target sites in tumor nodules to identify targeted TSG mutations and determine the frequencies and spectra of these mutations. We define an allelic mutation with a frequency at no less than 5% as a true mutation to reduce the interference of sequencing errors. While different targets showed different mutational spectra, we also found that many same target sites carried different allelic mutations with varying frequencies. For example, in analysis of a tumor nodule induced by *Sp*Cas9 together with 35 sgRNAs each at 0.8 mg, in addition to their respective wild-type (WT) allele, the *p53* target site had 3 types of mutations, 28.12% for 1-bp deletion on the left side of the break (termed Del1|0), 16.13% for 7-bp deletion on the right side of the break (Del0|7), and 13.87% for insertion of 1A at the break (Ins1A); *Atm* had two (23.81% for D6|0 and 20.07% for Ins1C), and *Rb1* had none in addition to WT alleles (Fig. 2A). In this tumor module, majority of target genes were mutated at intended sites and the mutation frequencies (MF) were high at some of these sites after the frequencies of all allelic mutations were combined for each site (Fig. 2B). This was further confirmed by analysis of more tumors induced by *Sp*Cas9 together with 35 sgRNAs each at 1 or 3 mg (Fig. 2C; Additional file 1: Table S2; Additional file 2: Fig. S3). The mutation profiles differed between tumors within a same mouse or from different mice, indicating intertumoral heterogeneity in CRISPR/Cas9-induced liver cancer with respect to targeted mutations of TSGs.

In addition, a tumor nodule from mouse #5 was microdissected into four regions, and each region analyzed for targeted TSG mutations by NGS of PCR-amplified target sites. Significant variations existed in mutation profiles between these four regions (Fig. 2C; Additional file 1: Table S2; Additional file 2: Fig. S3), suggesting ITH of targeted TSG mutations. These variations may be also attributable to varying healthy stromal components and/or residual normal tissue in individual tumor nodules.

To further analyze ITH of targeted TSG mutations, we established single-cell clones from tumor nodules 5T2, 6T1, 7T1, and 8T1 from different mice and analyzed targeted mutations in these tumor cells by targeted PCR amplicon deep sequencing (Additional file 2: Fig. S3). Intriguingly, approximately half of 34 TSGs were highly mutated between individual clones from the same nodules (in red in Fig. 2C). For some other targeted genes, e.g., *Smad4* and *Tsc1*, the frequencies of targeted mutations varied widely between 0 and 100% among single-cell clones from the same nodules, e.g., 1T1 (Fig. 2C). Between different clones from the same nodules, some targets sites carried identical mutations with similar frequencies but some harbored identical mutations with different frequencies or even different mutations (Fig. 2C; Additional file 1: Table S3). These results indicated strong ITH of targeted TSG mutations in CRISPR/Cas9-induced mouse liver cancer and suggested that single-cell clones originating from the same nodules undergo multiple edits by this 34-sgRNA library of TSGs.

As observed previously [22], individual target sites often carried more than two different allelic mutations within a tumor nodule (Additional file 1: Table S2). This could be explained at least by the following three possibilities. (1) Several transfected founder cells happened to start together with a different subset of mutations induced by multiplexed CRISPR/Cas9 genome editing and developed into a single tumor nodule. (2) Some mutations were first induced in a single transfected founder cell by multiplexed CRISPR/Cas9 genome editing and others occurred only after the first cell division in subsequent daughter or granddaughter cells [22]. (3) More than two copies of target sites exist in the genome of a single transfected founder liver cell due to polyploidization of liver cells [49–51] and are differently mutated by CRISPR/Cas9. However, in some single-cell clones (e.g., 1C3 and 6C7), a few target sites harbored more than two different allelic mutations and even up to 9 different allelic mutations, some of which occurred with varying frequencies (Additional file 1: Table S3). This was unexpected for a single-cell clone where only two copies of a target site are the most likely for CRISPR/Cas9-induced mutations unless the cells are hyperploid or the target sites have repeat sequences. Nevertheless, this type of copy number variations (CNVs) at the single-cell level represents a new source for heterogeneity of targeted TSG mutations in CRISPR/Cas9-induced mouse liver cancer.

### Type and frequency of targeted mutation alterations from parental clones to subclones

**Fig. 3:**
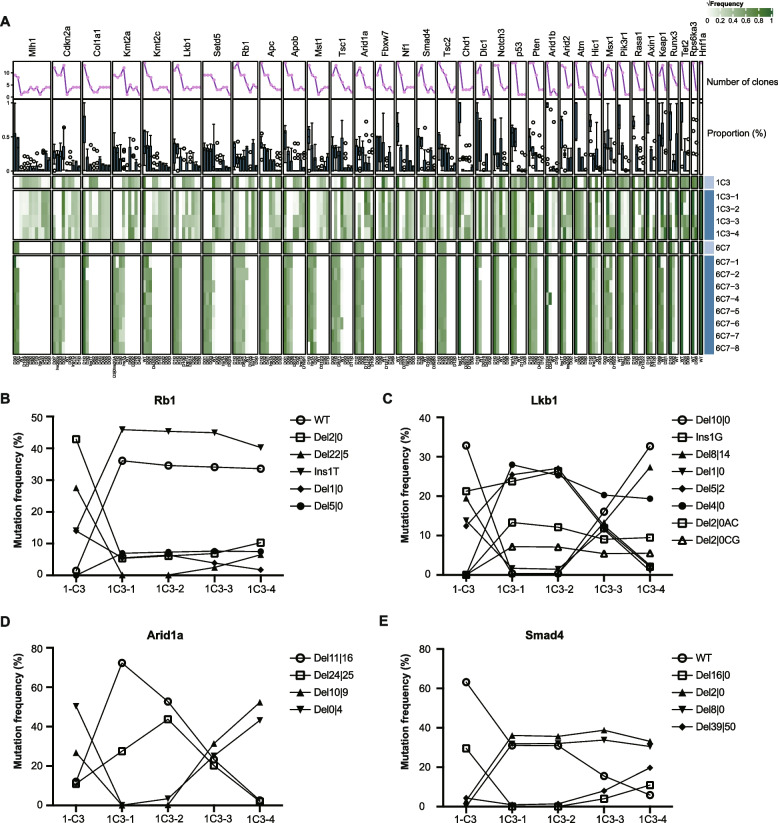
Alteration of target site mutations between subclones of single-cell clone 1C3 and 6C7 derived from mouse liver tumors. **A** Changes in target site mutation profiles between parental single-cell clones (i.e., 1C3 and 6C7) and their subclones (1C3-1, 1C3-2, 1C3-3, and 1C3-4 for 1C3 and 6C7-1, 6C7-2, 6C7-3, 6C7-4, 6C7-5, 6C7-6, 6C7-7, and 6C7-8 for 6C7). Gene targets and their mutation types are shown on top and at bottom, respectively. The numbers flanking Del or Ins indicated at bottom are the numbers of deleted or inserted nucleotides. √Frequency representing square root of mutation frequencies is indicated from the highest 1 in dark green to the lowest 0 in white. The top line chart and the boxplot under show the number of single-cell clones with a specific mutation type, i.e., number of clones, and the percentage distribution of a specific mutation type in all single-cell clones, i.e., proportion (%), respectively. **B**–**E** Representative target mutation oscillation of *Rb1* (**B**), *Lkb1* (**C**), *Arid1a* (**D**), and *Smad4* (**E**) between parental clone 1C3 and its subclones

Given the presence of more than two allelic mutations with significant different frequencies in a single-cell clone, we wondered whether this mutation pattern is inheritable. We thus selected two single-cell clones (i.e., 1C3 and 6C7) that carried more than two copies of allelic mutations at some target sites, isolated 4 subclones from 1C3 and 8 subclones from 6C7, and analyzed targeted mutations in these clones and subclones. The profile of target site mutations was different between parental clones and their respective subclones and between subclones (Fig. 3A; Additional file 1: Table S4). For instance, 6 *Rb1* target site variants, 8 *Lkb1* target site variants, 4 *Arid1a* target site variants, and 5 *Smad4* target site variants were detected across parental 1C3 clone and its 4 subclones, but each type displayed different frequencies within individual clones and the mutation profiles were also different between clones (Fig. 3B–E). The frequency of the wide type *Rb1* was less than 2% in paternal clone 1C3 but over 30% in 4 subclones (Fig. 3B). In contrast, the frequency of the *Rb1* Del22|5 mutation was nearly 30% in 1C3 but hardly detectable in subclone 1C3-1 or 1C3-2, less than 3% in 1C3-3 and about 5% in 1C3-4 (Fig. 3B). The *Lkb1* Del4|0 mutation was barely detected in 1C3, but the frequencies of this mutation were 20% or more in 4 subclones (Fig. 3C). The *Lkb1* Del10|0 mutation was a dominant mutation with the frequency at over 30% in 1C3 and its subclone 1C3-4 but less than 1% in subclone 1C3-1 and 1C3-2 and around 16% in subclone 1C3-3 (Fig. 3C). The *Arid1a* Del11|16 mutation was dominant at more than 70% in 1C3-1 but negligible at less than 2% in 1C3-4 (Fig. 3D). In contrast to Del11|16, the *Arid1a* Del10|9 mutation was nearly undetectable in 1C3-1 but highly frequent at more than 50% in 1C3-4 (Fig. 3D). Similarly, while the *Smad4* WT allele was detected at over 60% in 1C3 but less than 6% in 1C3-4, the frequency of the Smad4 Del2|0 was 2% in 1C3 but nearly 35% in 1C3-4 (Fig. 3E).

The 6C7 parental clone and its subclones were more similar to each other than 1C3 the 1C3 parental clone and its subclones to each other (Fig. 3A; Additional file 1: Table S4). Still, while the *Rb1* WT allele in 6C7-2 was over 15%, the other 6C7 subclones as well as the parental clone carried infrequent WT allele of *Rb1* (Additional file 2: Fig. S4A). Similarly, the frequency of *Lkb1* Del1|0 mutation in 6C7 consistently matched that of its subclones, except for 6C7-1, which displayed a higher mutation frequency (Additional file 2: Fig. S4B). Additionally, the frequencies of three *Arid1a* target mutation variants oscillated among the 6C7 parental clone and its subclones (Additional file 2: Fig. S4C) and a change in the *Smad4* Del2|0 and Del1|0 frequency were observed in 6C7-7 as compared to the 6C7 parental clone and the other subclones (Additional file 2: Fig. S4D). These results together indicate continuing oscillation in the frequencies of target site mutations between these parental clones and subclones. In particular, predominant mutations in parental clones could disappear in subclones whereas negligible mutations in parental clones could appear in subclones.

As spontaneous mutations at a given site normally occur with extremely low probability during cell proliferation [52], it is surprising that the frequency of a specific targeted mutation oscillates significantly from parental single-cell clones to daughter single-cell subclones. We speculated that Cas9-sgRNA might be stably expressed in clones isolated and continue to edit the WT target sites, thus altering the frequency of the targeted mutation. However, we did not detect *Sp*Cas9 proteins in the parental clones 1C3 and 6C7 and their subclones (Additional file 2: Fig. S5A). It remains possible that a small amount of *Sp*Cas9 proteins stably synthesized in the cells could actively mutate their target sites, even if the protein level is hardly detectable by Western blot. We thus transfected these parental clones or subclones with the expression plasmids for two *Col1a1* sgRNAs and one *Rosa26* sgRNA with or without the *Sp*Cas9 expression plasmid and measured the editing frequencies at the target sites by PCR amplicon deep sequencing. While the editing is efficient with transfection of both *Sp*Cas9 and sgRNA, no editing was detected with sgRNA transfection alone or with neither *Sp*Cas9 nor sgRNA (Additional file 2: Fig. S5B-C). This excludes the possibility that continuing CRISPR/Cas9 genome editing alters the types and frequencies of targeted TSG mutations from parental clones to subclones.

### Continuing type and frequency oscillation of targeted mutations during clonal expansion

**Fig. 4:**
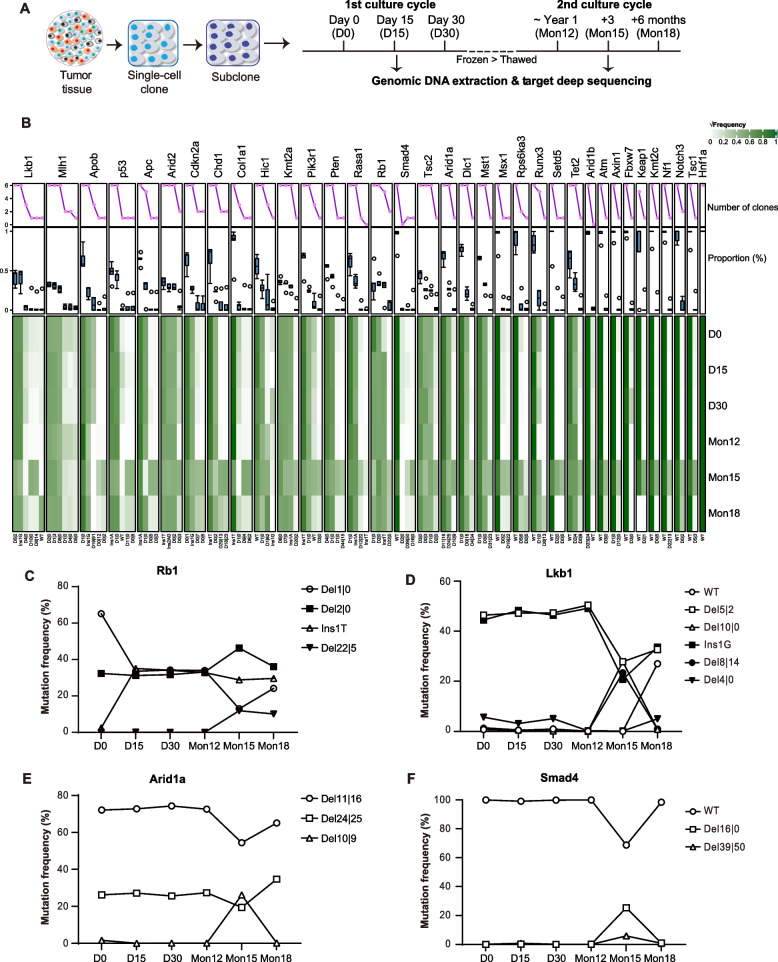
Oscillation of target site mutations during proliferation of the single-cell subclone 1C3-1 derived from 1C3. **A** Schematic of experimental outline. Proliferation of the single-cell clone 1C3-1 was divided the first culture cycle that continues for 30 days and the second culture cycle that continues after 1 year storage. Target site mutations were determined by targeted PCR amplicon deep sequencing of gDNA extracted from cells at different time points of cell proliferation as indicated. D0, day 0; D15, day 15; D30, day 30; Mon12, year 1; Mon15, year 1 + 3 months; Mon12, year 1 + 6 months. **B** Changes in target site mutation profiles of 1C3-1 at indicated time points of cell proliferation (i.e., days 0, 15, and 30 and Mon12, Mon15, and Mon18). Gene targets and their mutation types are shown on top and at bottom, respectively. The numbers flanking Del or Ins indicated at bottom are the numbers of deleted or inserted nucleotides. √Frequency representing square root of mutation frequencies is indicated from the highest 1 in dark green to the lowest 0 in white. The top line chart and the boxplot under show the number of single-cell clones with a specific mutation type, i.e., number of clones, and the percentage distribution of a specific mutation type in all single-cell clones, i.e., proportion (%), respectively. **C**–**F** Representative target mutation oscillation of *Rb1* (**C**), *Lkb1* (**D**), *Arid1a* (**E**), and *Smad4* (**F**) during cell proliferation of 1C3-1

Next, we asked whether the types and frequencies of targeted mutations change over time during expansion of a subclone. We continuously cultured 4 subclones (i.e., 1C3-1, 1C3-2, 1C3-3 and 1C3-4) and analyzed target site mutations of each subclone on day 0, day 15, and day 30 (Fig. 4A; Additional file 2: Fig. S6). The frequencies of some targeted mutation changed over time during clonal expansion, e.g., from 2.47% at day 0 to 35.09% at day 15 and 34.09% at day 30 for Rb1 Ins1T in 1C3-1 (Additional file 1: Table S5; Additional file 2: Fig. S6). After storage in liquid nitrogen for a year, we thawed and continued to culture the 1C3-1 clone. We repeated analysis of target site mutations in the cultured cells on day 0 (i.e., Mon12), day 90 (i.e., Mon15), and day 180 (i.e., Mon18) (Fig. 4A). The frequencies of some targeted mutations continued to change over time during cell culturing of 1C3-1 (Fig. 4B; Additional file 1: Table S5). For example, the *Rb1* Ins1T mutation was negligible on day 0 but appeared with the frequencies at over 30% on day 15 and day 30. Del1|0 mutation was significant at over 60% on day 0 and decreased to about 30% on day 15, day 30, and Mon12 and even 10% on Mon15. On Mon18, this mutation was slightly increased to over 20% (Fig. 4C). Both *Lkb1* Del5|2 and Ins1G mutations remained frequent at over 40% from day 0 to Mon12 but decreased to about 20% on Mon15 and increased again to 30% on Mon18 (Fig. 4D). *Arid1a* Del11|16 and Del10|9 mutations changed in opposite direction on Mon15 and Mon18 (Fig. 4E). Similarly, *Smad4* WT and Del16|0 mutations oscillated in opposite direction on Mon15 and Mon18 (Fig. 4F). This data further indicates that some targeted mutations are not stable during proliferation of single tumor cells derived from CRISPR/Cas9-induced liver cancer in mice.

### Type and frequency alteration of targeted mutations from single-cell clones to subcutaneous grafts

**Fig. 5:**
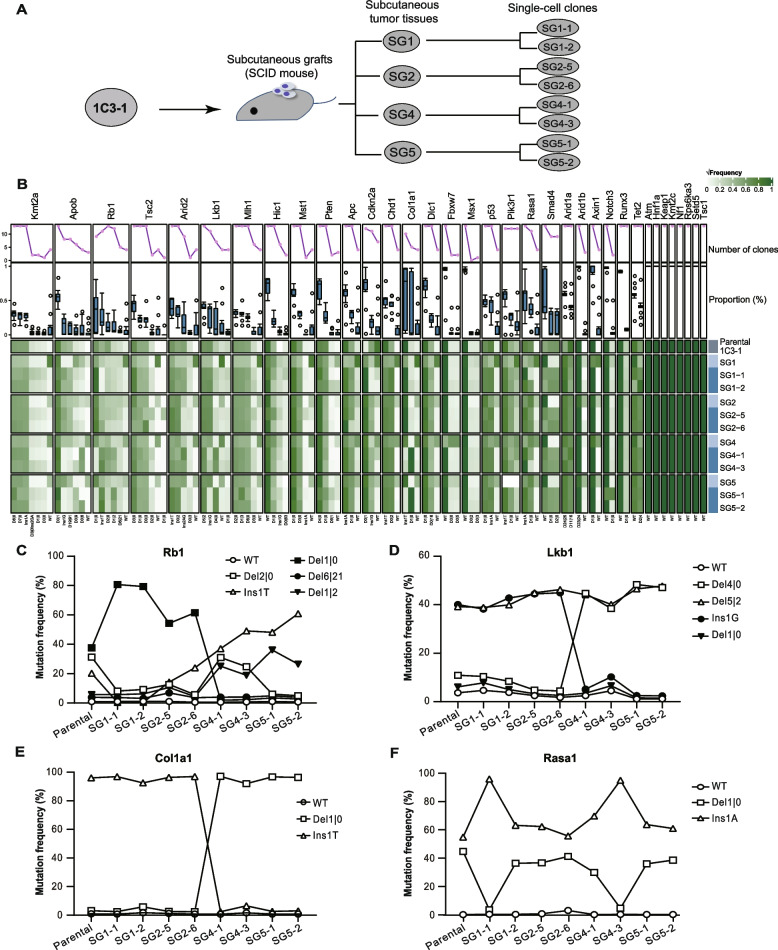
Oscillation of target site mutations during subcutaneous tumor cell grafts of 1C3-1. **A** Schematic of experimental outline. After subcutaneous implantation of the single-cell subclone 1C3-1 into SCID mice, tumors were formed and four tumor tissues (SG1, SG2, SG4, and SG5) from different mice collected at 2 weeks post inoculation. Two single-cell clones were derived from each of these four tumor tissues as indicated. Target site mutations were determined by targeted PCR amplicon deep sequencing of gDNA extracted from cells. SG, subcutaneous graft. **B** Changes in target site mutation profiles of single-cell clones from subcutaneous tumor cell grafts as indicated. Gene targets and their mutation types are shown on top and at bottom, respectively. The numbers flanking Del or Ins indicated at bottom are the numbers of deleted or inserted nucleotides. √Frequency representing square root of mutation frequencies is indicated from the highest 1 in dark green to the lowest 0 in white. The top line chart and the boxplot under show the number of single-cell clones with a specific mutation type, i.e., number of clones, and the percentage distribution of a specific mutation type in all single-cell clones, i.e., proportion (%), respectively. **C**–**F** Representative target mutation oscillation of *Rb1* (**C**), *Lkb1* (**D**), *Col1a1* (**E**), and *Rasa1* (**F**) across single-cell clones derived from subcutaneous tumor cell grafts

In order to evaluate this instability of targeted TSG mutations in vivo, we implanted the subclone 1C3-1 cells into 4 immunodeficient SCID mice subcutaneously (Fig. 5A). All mice grew a visible tumor in about 5 days. We harvested the tumor tissues from these 4 mice at the 14th day and established 2 single tumor cell clones from each tumor tissue (Fig. 5A). Analysis of targeted mutations revealed that tumor tissues harbored more frequent WT allele than both 1C3-1 and tumor cell clones derived from tumor tissues, likely due to the presence of normal cells in subcutaneous tumor samples (Additional file 1: Table S6). Therefore, in order to exclude the interference of normal cells from tumor tissues on the frequency of targeted mutations in tumor cells, we compared the frequencies of targeted mutations only between parental 1C3-1 clone and tumor cell clones (Fig. 5B). The frequencies of some targeted mutations changed significantly among these tumor cells (Fig. 5B; Additional file 1: Table S6). For instance, 1 bp deletion (Del1|0) of *Rb1* was dominant with the frequencies at over 50% in SG1-1, SG1-2, SG2-5, and SG2-6 but infrequent at less than 5% in SG4-1, SG4-3, SG5-1, and SG5-2 (Fig. 5C). Differently, the parental subclone 1C3-1 harbored this *Rb1* Del1|0 mutation at 40% (Fig. 5C). Furthermore, while the Del5|2 mutation of *Lkb1* changed little with the frequency at about 40% among all tumor cells tested, the *Lkb1* Ins1G mutation oscillated significantly among these tumor cells, with the frequency at about 40% in SG1-1, SG1-2, SG2-5, and SG2-6 as well as 1C3-1 but nearly undetectable in SG4-1, SG4-3, SG5-1, and SG5-2 (Fig. 5D). In addition, two *Col1a1* mutations, i.e., Del1|0 and Ins1T, oscillated in an opposite direction (Fig. 5E). While the *Col1a1* Del1|0 mutation dominant in 1C3-1, SG1-1, SG1-2, SG2-5, and SG2-6 was negligible in SG4-1, SG4-3, SG5-1, and SG5-2, the Col1a1 Ins1T mutation infrequent in 1C3-1, SG1-1, SG1-2, SG2-5, and SG2-6 occurred frequently in SG4-1, SG4-3, SG5-1, and SG5-2 (Fig. 5E). Similarly, the Del1|0 and Ins1A mutations of *Rasa1* started with the frequencies at around 50% in parental 1C3-1 clone and then oscillated in a reverse pattern among single-cell clones from tumor grafts (Fig. 5F). These results indicate that targeted mutations in tumor cells derived from CRISPR/Cas9-induced liver cancer in mice are also unstable in subcutaneous grafts derived from tumor cells with the frequencies of some targeted mutations oscillating in vivo.

### Increased genomic instability in CRISPR/Cas9-induced mouse liver cancer cells

**Fig. 6:**
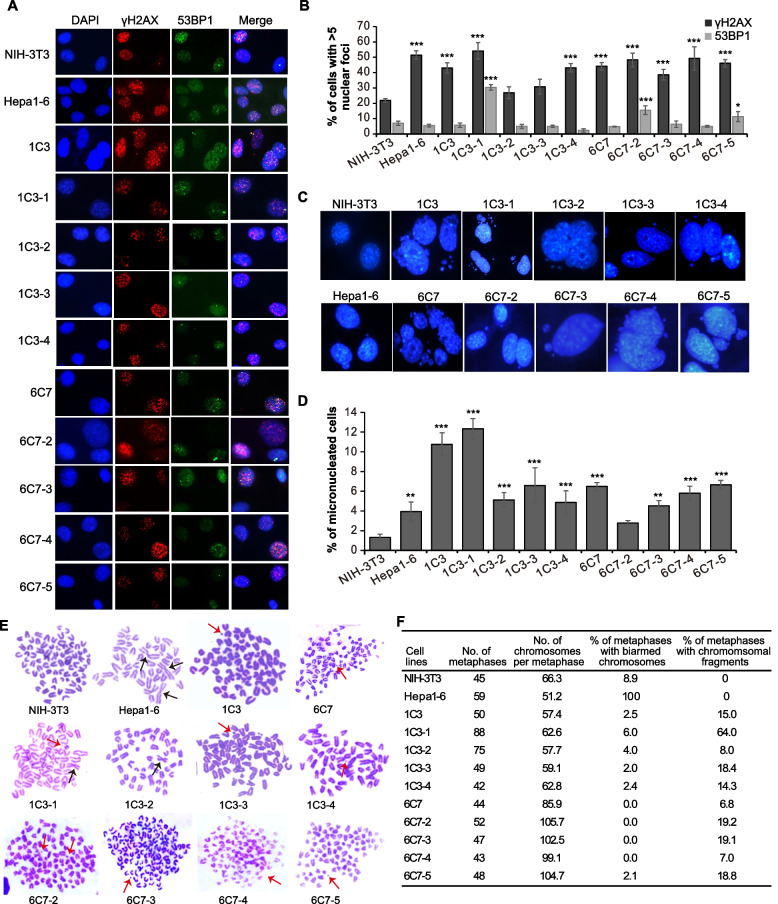
Genomic instability in single-cell clones derived from CRISPR/Cas9-induced mouse liver tumors. **A** Microscopic images of spontaneous γH2AX and 53BP1 foci in primary mouse liver cancer cells. **B** Percentage of cells with > 5 γH2AX foci or 53BP1 foci. Columns indicate the mean ± S.E.M from three independent experiments with statistical significance detected by One-way ANOVA with Dunnett’s multiple comparison test (vs. NIH-3T3). *P < 0.05 and ***P < 0.001. **C** Microscopic images of micronuclei (MN) in primary mouse liver cancer cells. **D** Percentage of cells with micronuclei. Columns indicate the mean ± S.E.M from three independent experiments with statistical significance detected by One-way ANOVA with Dunnett’s multiple comparison test (vs. NIH-3T3). **P < 0.01 and ***P < 0.001. **E** Representative images of chromosomal aberrations in metaphase spread of primary mouse liver cancer cell lines. Red arrows and black arrows indicate chromosomal fragments and biarmed chromosomes, respectively. **F** Summary of metaphase spread analysis. In each indicated cell line, the number of metaphases, number of chromosomes per metaphase, and percentages of metaphases with biarmed chromosomes and with chromosomal fragments are calculated and shown

As mentioned previously [52], spontaneous mutations at a given site normally occur at an extremely low rate and are impossible to cause significant alterations in the types and frequencies of targeted mutations detected in the study. Continuing targeted editing by Cas9-sgRNA was also excluded as a causal factor because neither stable expression nor the editing activity of Cas9-sgRNA was detected in these single-cell clones. As cancer with genomic instability has an increased tendency for constant genomic alteration, we wondered whether these primary liver tumor cells are associated with strong genomic instability. We first examined spontaneous γ-H2AX and 53BP1 foci formation in 10 primary tumor cell lines derived from CRISPR/Cas9-induced mouse liver cancer, the control cell line NIH3T3, and the mouse liver cancer cell line Hepa1-6 (Fig. 6A). All of these liver cancer cell lines except the 1C3-2 clone showed a higher level of spontaneous γH2AX focus formation than the control NIH3T3 cells (Fig. 6A–B), indicating strong induction of spontaneous DNA DSBs and activation of DNA damage response in these cancer cells. It was however unclear why 53BP1 focus formation was much less frequent than γH2AX focus formation in nearly all of these cell lines as both γH2AX and 53BP1 foci indicated the site of DSBs (Fig. 6A–B).

We also analyzed these CRISPR/Cas9-induced primary liver tumor cells for micronucleus formation, which is frequently involved in chromosomal aberrations and genomic instability in cancer [53]. Micronucleus formation was readily detected in these tumor cells (Fig. 6C). The percentages of micronucleated cells were significantly higher in tumor cells than in the control NIH3T3 cells (Fig. 6D). In particular, while about 1% of NIH3T3 cells contained micronuclei, over 10% of 1C3 or 1C3-1 cells were micronucleated (Fig. 6D). Metaphase spread analysis further identified significant chromosomal aberrations in primary liver tumor cells (Fig. 6E). The number of chromosomes in primary liver tumor cells varied in average from 57.4 in 1C3 to 105.7 in 6C7-2 and was much more than 40 in a diploid mouse cell (Fig. 6E–F). This indicates that these tumor cells are hyperploid, at least in part contributing to more than two allelic variations at a target site of TSGs in single-cell clones. Biarmed chromosomes appeared in all Hepa1-6 cells as reported previously [54], but only existed in 2.0–6.0% of the primary liver tumor cell lines 1C3, all 1C3 subclones, and 6C7-5 as well as in 8.9% of NIH-3T3 (Fig. 6E–F). In contrast, we did not detect any biarmed chromosomes in 6C7 or its subclones 6C7-2, 6C7-3, and 6C7-4. The difference in the generation of biarmed chromosomes between 6C7-5 and its parental clone 6C7 or other 6C7 subclones indicates potential variations in genomic instability in these cells (Fig. 6E–F). In addition, primary liver tumor cells exhibited more frequent chromosomal fragments than NIH-3T3 and Hepa1-6 (Fig. 6E–F). As frequent micronucleus formation, chromosomal aberrations, and chromosomal fragments in primary tumor cells manifested the genomic instability of CRISPR/Cas9-induced mouse liver tumors, these findings indicated a possible connection between the genomic instability and the oscillation in the frequencies of targeted TSG mutations in tumor cells. Variations in genomic instability among parental clones (i.e., 1C3 and 6C7) and their respective subclones again implied that an intrinsic genetic force drive the oscillation of target site mutations in these single-cell clones.

### Detectable eccDNA harboring targeted site mutations

Due to unstable nature, linear chromosomal fragments observed in our study could drive significant alterations in the frequencies of targeted mutations in tumor cells derived from CRISPR/Cas9-induced mouse liver cancer. However, it was technically difficult to separate linear chromosomal fragments from intact chromosomes for sequencing. Thus, we were unable to determine the extent to which linear chromosomal fragments contributed to the oscillation in the frequencies of targeted mutations in tumor cells. Like linear chromosomal fragments, eccDNA could be formed in CRISPR/Cas9-induced mouse liver cancer; however, as eccDNA could be separated from gDNA and identified by Circle-Seq [45], it was possible to determine the contribution of eccDNAs in the oscillation of the targeted TSG mutation frequencies in tumor cells.

**Fig. 7:**
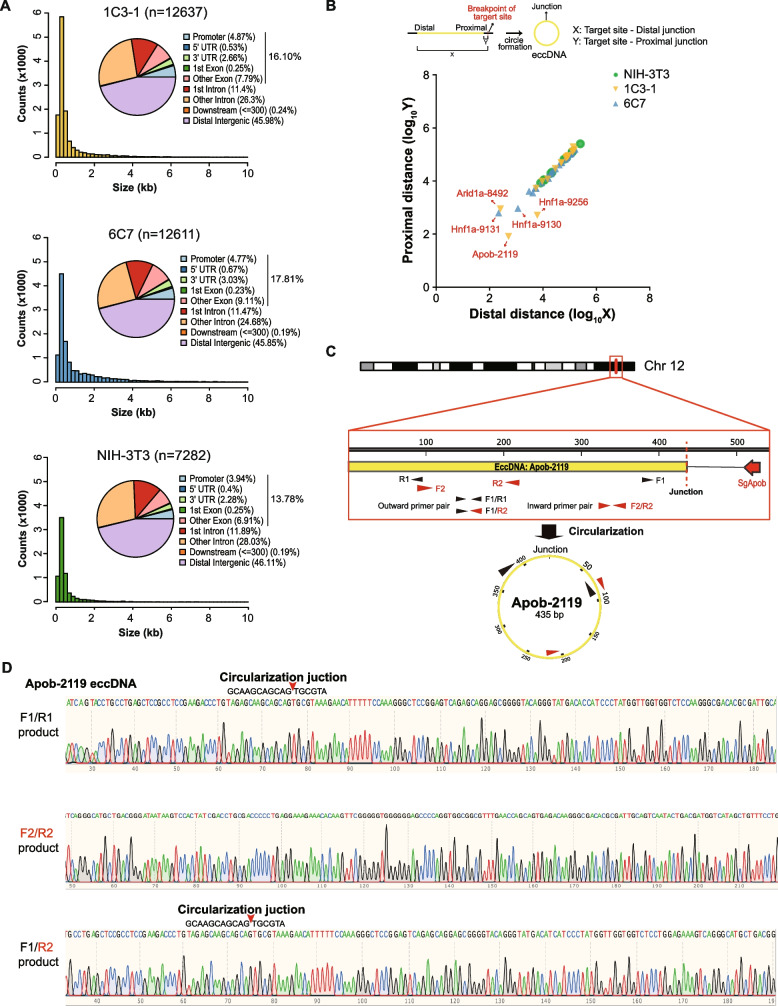
Identification and mapping of eccDNAs derived from the single-cell clone 1C3-1 and 6C7 by Circle-Seq. **A** EccDNA size distribution and mapping of eccDNAs to genic and intergenic regions in 1C3-1 (top) and 6C7 (middle) as well as in the control NHH-3T3 cell line (bottom). Total number of eccDNAs are indicated in parentheses next to the name of the cell line. Pie charts depict the distribution and percentage of eccDNAs mapped to different genic and intergenic regions in the three cell lines. Percentages of eccDNAs are also shown for genic regions that include promoters, exons, 5′-UTR and 3′-UTR. **B** Distribution of eccDNAs neighboring TSG target sites in various distances indicated by log_10_ values from distal and proximal junction point to TSG target sites in NIH-3T3, 1C3-1, and 6C7 cell lines as indicated. EccDNAs that are close to TSG target sites are also denoted by arrows and names. X and Y in the schematic (top) represent the distance from the distal and proximal junction point of eccDNAs on the chromosome to the breakpoint of TSG target site by CRISPR/Cas9, respectively. **C** Schematic for circularization of *Apob*-2119 eccDNA from chromosome 12 (Chr 12). Likely due to cleavage of the *Apob* target site by CRISPR/Cas9, the left end was processed and circularized via neighboring microhomology (MH) in the same chromosome to form *Apob*-2119 eccDNA. The four primers F1, R1, F2, and R2 indicated were designed to form one pair of inward primers F2/R2 and two pairs of outward primers R1/F1 and R2/F1 for validation of *Apob*-2119 eccDNA. The length of the eccDNA and the position of *Apob* target site by CRISPR/Cas9 are shown with the distal point of the eccDNA set at 0 bp. **D** Sequences of *Apob*-2119 eccDNA and its junctions as shown are determined by PCR with indicated primer pairs followed by Sanger sequencing. Circularization junction is also indicated

We thus isolated eccDNAs from the 1C3-1 clone and the 6C7 clone along with the control NIH3T3 cells, enriched eccDNAs by removal of linear DNA with Plasmid-Safe ATP-Dependent DNase and removal of mitochondrial DNA with PacI and RCA of eccDNAs with Phi29 DNA polymerase, and performed Circle-Seq followed by Circle-MAP (Additional file 2: Fig. S7A). Removal of linear DNA by Plasmid-Safe ATP-Dependent DNase and mitochondrial DNA by PacI from eccDNAs was confirmed by targeted PCR amplification of the nuclear gene *Actb* and *Cox5b* and the mitochondrial gene *mt-Co1* (Additional file 2: Fig. S7B-D). Circle-MAP revealed that the numbers of eccDNAs were respectively 12,637 in 1C3-1 and 12,611 in 6C7, greater than 7282 in NIH3T3 (Fig. 7A; Additional file 1: Table S7). The length distribution of eccDNAs was similar in all three cell lines, and the majority of these eccDNAs were small in size (Fig. 7A). For example, the length of eccDNA ranged from 150 to 9993 bp with the median length of 344 bp in the 1C3-1 clone, from 150 to 9962 bp with the median length of 466 bp in the 6C7 clone, and from 150 to 9987 bp with the median length of 333 bp in the control NIH3T3 cells (Fig. 7A); 83%, 68%, and 86% were less than 1000 bp and 91%, 82%, and 93% less than 2000 bp in 1C3-1, 6C7, and NIH3T3, respectively (Fig. 7A; Additional file 1: Table S7). Additionally, eccDNAs were mostly distributed in distal intergenic region and introns; however, the distribution of eccDNAs in genic regions such as promoters, 5′-UTRs, 3′-UTRs, and exons was more frequent in 1C3-1 and 6C7 cells than in NIH3T3 cells (i.e., 15.78% in 1C3-1 and 17.81% in 6C7 vs. 13.78% in NIH3T3).

We also mapped eccDNAs to 24 chromosomes and determined the chromosomal regions from which each eccDNA was likely originated (Additional file 1: Table S7; Additional file 2: Fig. S8A). After selecting eccDNAs that were matched to target TSG regions as well as the *Setd5* control (Additional file 1: Table S7; Additional file 2: Fig. S8B-D), we analyzed the distance from the breakpoints at Cas9-sgRNA target sites of TSGs and *Setd5* to the sites that were corresponding to the distal and proximal junction of eccDNAs. The distance was generally shorter in the 1C3-1 clone and the 6C7 clone than in the control NIH3T3 cells (Fig. 7B; Additional file 1: Table S7). Among eccDNAs near target TSGs, the 435-bp Apob-2119 eccDNA near Apob was the closest eccDNA to a target TSG in 1C3-1 cells (Fig. 7B; Additional file 1: Table S7). To further confirm generation of Apob-2119 eccDNA, we performed outward PCR with primer pair F1/R1 and primer pair F1/R2 to amply circularization junction of Apob-2119 eccDNA and inward PCR with primer pair F2/R2 to amply part of Apob-2119 eccDNA sequence after eccDNA purification (Fig. 7C; Additional file 2: Fig. S9A). No PCR bands less than 435 bp were detected in the control NIH3T3 cells and the 6C7 clone; however, PCR bands less than 435 bp were clearly generated from eccDNAs in the 1C3-1 clone (Additional file 2: Fig. S9B). The sequences of PCR products less than 435 bp from outward and inward primer pairs were perfectly aligned with the Apob-2119 eccDNA (Fig. 7D).

However, eccDNAs identified by Circle-Seq harbored no targeted TSG mutations. If eccDNAs harboring targeted TSG mutations were in a trace amount, Circle-Seq might not be sensitive enough to detect such eccDNAs. In contrast, PCR amplification of targeted TSG mutations in eccDNAs, followed by next-generation sequencing, could allow detection of eccDNAs that harbored targeted TSG mutations, even in a trace amount (Additional file 2: Fig. S10A). Indeed, using eccDNAs isolated from 1C3-1, 1C3-2, 1C3-3, 6C7-2, and 6C7-4 clones as the template for targeted PCR, we found that many of 35 target sites were poorly amplified as compared to gDNA and the numbers of deep sequencing reads for these sites were low, indicating the absence of eccDNA harboring these sites (Additional file 1: Table S8; Additional file 2: Fig. S10B). Only 13 targets (i.e., *Atm*, *Cdkn2a*, *Col1a1*, *Fbxw7*, *Kmt2c*, *Nf1*, *Notch3*, *p53*, *Pik3r1*, *Rb1*, *Rps6ka3*, *Setd5*, and *Tet2*) consistently exhibited sufficient reads in eccDNAs (Additional file 2: Fig. S10B and Fig. S10). Deep sequencing of targeted amplicons further confirmed that eccDNA in 1C3-1, 1C3-2, 1C3-3, 6C7-2, and 6C7-4 clones contain intact TSG target sites and targeted TSG mutations (Additional file 1: Table S6; Additional file 2: Fig. S11).

**Fig. 8:**
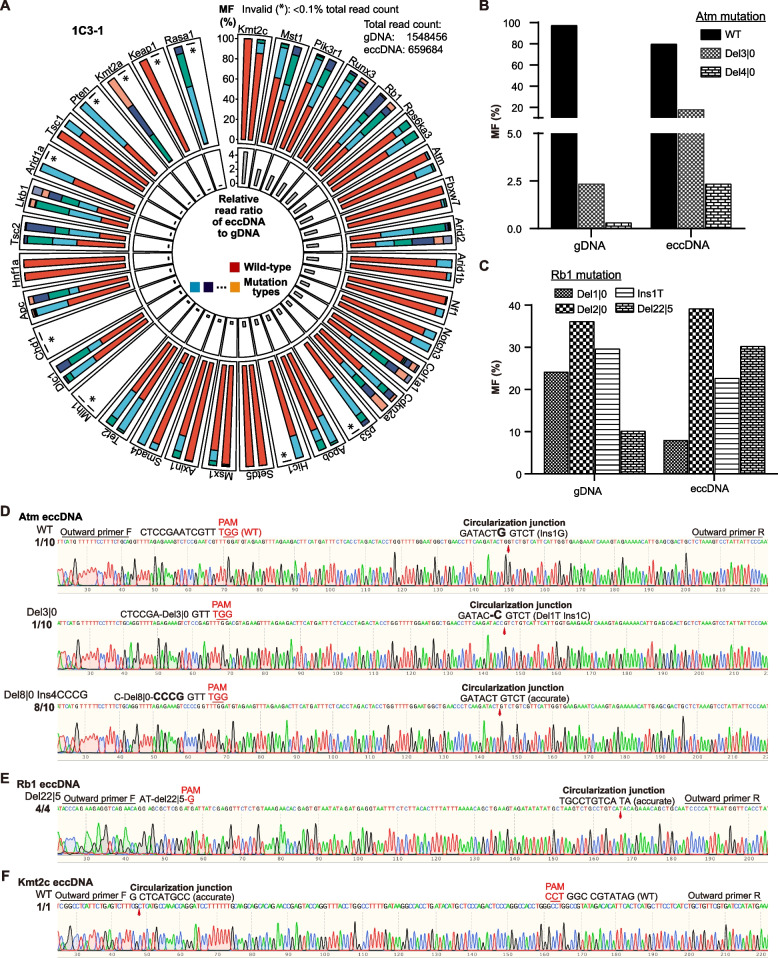
Target site mutations of TSGs detected on eccDNA. **A** Circular barplot for target site mutations of 35 targets in gDNA and eccDNA in representative single-cell clone 1C3-1. The column in the inner circle for each target gene were relative read ratios of eccDNA to gDNA for each target site from deep sequencing data. The stacked barplot in the outer circle represents the mutation pattern at indicated target sites in eccDNA and gDNA. Mutation types are shown in color. Total read counts of eccDNA and gDNA are listed. *, invalid read counts at indicated sites, which are defined as < 0.1% of total read counts from eccDNA or gDNA. **B**, **C** Target site mutation distribution of representative TSGs *Atm* (**B**) and *Rb1* (**C**) in gDNA and eccDNA. **D**–**F** Circularization junctions of eccDNA containing target sites for *Atm* (**D**), *Rb1* (**E**), and *Kmt2c* (**F**). Circularization junction sequences as shown are determined by PCR with a pair of indicated outward primers followed by Sanger sequencing. Target sites with PAM and mutations are also indicated

To compare the distribution of targeted mutations across 34 target sites between eccDNA and gDNA, we calculated percentages of combined reads from all 34 target sites for reads of each target site respectively in both eccDNA and gDNA and derived relative read ratio of eccDNA to gDNA for each target site in the 1C3-1 clone by dividing percentage values for eccDNA to respective ones for gDNA (Additional file 2: Fig. S10C-D). Because PCR variations of eccDNA were normalized by PCR variations of gDNA in this relative read ratio of eccDNA to gDNA, the interference of PCR variations on sequencing reads in eccDNA across target sites were partly excluded. While the profiles of targeted mutations were different between eccDNA and gDNA as expected, it was surprising that some mutations undetectable or with a low frequency in gDNA increased significantly in frequency in eccDNA (Fig. 8A; Additional file 2: Fig. S10C-D and Fig. S11). For example, the frequency of *Atm* Del3|0 mutation was less than 2.35% in gDNA but was elevated to 17.88% in eccDNA (Fig. 8B). Similarly, the frequency of the *Rb1* Del22|5 mutation is 9.95% in gDNA but increased to 28.53% in eccDNA (Fig. 7C). In addition, some targeted mutations detected at a high rate in gDNA were undetectable or much less frequent in eccDNA harboring these targets (Fig. 8A; Additional file 1: Table S8; Additional file 2: Fig. S10D and Fig. S11). For instance, the frequency of the *Rb1* Del1|0 mutation was 23.82% in gDNA but was lowered to 7.53% in eccDNA (Fig. 8C).

To directly identify eccDNA harboring targeted mutations, we designed outward PCR primer pairs to determine circularization junctions of eccDNA carrying the respective targets whereas inward primer pairs were usually used to identify targeted mutations (Additional file 2: Fig. S12A). Because 13 targets were frequently found in eccDNA isolated from 1C3-1 cells, we followed these 13 sites to design outward PCR primer pairs and identify circularization junctions of eccDNA. Because the outward PCR primer pairs are positioned at one side to targeted sites, target sites with or without any variations could also be identified (Additional file 2: Fig. S12A). Some of these 13 targets generated PCR bands with outward primer pairs, and PCR products with outward primer pairs were purified and cloned into a pUC19 vector for Sanger sequencing. We detected the circularization junctions of eccDNA harboring either intact target site or targeted mutations of *Atm*, *Rb1*, and *Kmt2c* (i.e., *Atm*, *Rb1*, and *Kmt2c* eccDNA) (Fig. 8D–F; Additional file 2: Fig. S12B). Based on positions of circularization junctions and targeted mutations, we determined that the sizes of *Atm*, *Rb1*, and *Kmt2c* eccDNA were about 200 bp, 211 bp, and 213 bp, respectively (Fig. 8D–F). Among 10 clones sequenced for *Atm* on eccDNA, 1 contained the WT sequence at the target site and Ins1G at the circularization junction, 1 contained Del3|0 at the target site and Del1T Ins1C at the circularization junction, and 8 contained Del8|0 Ins4CCCG at the target site and no deletion/insertion at the circularization junction (Fig. 8D). Among 4 clones sequenced for *Rb1* on eccDNA, all had Del22|5 at the target site and were joined accurately at the circularization junction, whereas 1 clone from *Kmt2c* eccDNA had WT target site and no deletion/insertion at the circularization junction (Fig. 8E–F). Most of these variations such as *Atm* WT, *Atm* Del3|0, *Rb1* Del22|5, and *Kmt2c* WT were also present in gDNA (Fig. 8B–C; Additional file 1: Table S8). Taken together, these results demonstrated the existence of eccDNA harboring some targeted mutations of TSGs in CRISPR/Cas9-induced mouse liver cancer. As eccDNA is unstable in varying copies and could be degraded or integrated back to regular chromosomes [40], eccDNA harboring targeted mutations may provide genetic materials to alter the frequencies of targeted mutations in the genome, driving ITH in CRISPR/Cas9-induced mouse liver cancer.

## Discussion

CRISPR/Cas9-based targeted somatic gene editing of TSGs has been used to induce HCC in mice [19–24], providing a useful tool to study the biology of HCC, one of the leading causes of cancer-related death worldwide. A few combinations of TSGs are identified to initiate liver tumors and even ITH of the tumors in mice [19–24]. By analyzing targeted mutations of TSGs, our work describes a characterization of IGH in mouse model of liver cancer induced by multiplexed CRISPR/Cas9. Specifically, our data show the following: (1) strong heterogeneity with respect to the types and frequencies of targeted TSG mutations between tumor nodules and within a tumor nodule; (2) significant variations in allelic mutation type and frequency at a TSG target site between single-cell clones; (3) more than two types of allelic variations with varied frequencies at many of TSG target sites within a single-cell clone; (4) oscillation of allelic variations in type and frequency at some of TSG target sites in subclones, during proliferation of a subclone, and in mouse subcutaneous graft of a subclone. In addition, these tumor cells exhibited strong genomic instability manifested by micronucleation, chromosomal aberrations, hyperploidy, and generation of eccDNAs. In particular, while polyploidization is a characteristic feature of hepatocytes [49–51], hyperploid liver tumor cells and altered hyperploidy could change the copy number of genes including targeted TSGs during cell division. In the meantime, eccDNA could cause CNVs by random integration into a chromosome during cell proliferation [7, 28, 30–32]. This may help explain persistent oscillation of allelic TSG mutation types and frequencies in single-cell clones. Together, this study identifies a potential source for IGH in mouse liver cancer induced by multiplexed CRISPR/Cas9.

Since being first discovered as double minutes (DMs) in 1965 [55], eccDNA have recently emerged as important genetic molecules, whose functions are implicated in normal cellular function and the development of human diseases including cancers [28, 30–32, 40]. However, how eccDNA is generated in mammalian cells remains elusive although this question has been under intensive investigation. Many mechanisms such as the breakage-fusion-bridge (BFB) cycle, micronucleation, chromothripsis, and cell death-induced DNA fragmentation have been proposed to explain the biogenesis of eccDNA and may involve DNA breakage and repair [38, 56–61]. The BFB cycle caused by chromosomal fusion is regarded as an initiating factor for a cascade of events such as micronucleation, chromothripsis, and even cell death, thus generating eccDNA [59, 62]. In micronuclei, chromosomes are often susceptible to DNA breakage due to incomplete nuclear envelope, leading to chromothripsis, a single catastrophic event where a chromosomal region is shattered into a number of fragments [8, 60]. While re-integration of micronuclei into primary nuclei during mitosis could allow DNA fragments to engage complex chromosomal rearrangements in the genome, some DNA fragments are circularized to form eccDNA. Similar to eccDNA generation, reintegration of eccDNA elements to the genome may also require DNA damage and DNA recombination although the underlying mechanisms are not well understood [7, 30–32, 60, 61]. EccDNA could also result from apoptotic DNA fragmentation and may enter neighboring cells to induce an innate immune response [38]. Given that tumor cells from mouse liver cancer model contained high levels of micronuclei and chromosomal aberrations as well as detectable eccDNAs, it is likely that chromosomal fusion, the BFB cycle and chromothripsis also occur, but this likelihood has yet to be confirmed. Our data have also shown that tumor cells are hyperploid in mouse liver cancer model induced by multiplexed CRISPR/Cas9. As chromothripsis can be promoted by hyperploidy [63], this suggests an additional mechanism of eccDNA generation.

Recent studies have identified oncogene-containing eccDNA and demonstrated that oncogenes on eccDNA are commonly amplified in cancer [29–35]. Due to usual lack of centromeres and unequal segregation, eccDNA can also drive the development of IGH [7, 28, 30–32]. Such eccDNA can markedly increase oncogene copy number, promote accessible chromatin and high oncogene expression, drive IGH, and accelerate tumor evolution. However, extra copies of TSGs on eccDNAs and their inactivating mutations were not expected to add more cancer-promoting capacity as a driver mutation than inactivating mutations on genomic TSGs. Thus, eccDNAs harboring TSG mutations, particularly small eccDNAs, are often ignored although they may support the development of ITH in punctuated tumor evolution. It is yet to be determined whether eccDNAs harboring TSG mutations are present or function in human cancer cells and, if so, what their functions are.

Previous study has indicated that the use of multiplexed CRISPR/Cas9 could simultaneously generate many on-target and off-target DSBs in a cell, leading to fragmented chromosomes, micronuclei formation, and undesired chromosomal rearrangements including insertions/deletions, translocations, duplications, and complex rearrangements [22, 26]. Even one single DSB induced by CRISPR/Cas9 is capable of initiating the BFB cycle, consequently leading to micronucleation, chromothripsis, aneuploidy, or cell death [62]. Chromosomal fragments generated either directly from DNA cutting by multiplexed CRISPR/Cas9 or indirectly from chromothripsis or cell death could be circularized by DNA ligases, forming eccDNA [38, 61]. Even linear chromosomal fragments generated by paired CRISPR/Cas9 could form eccDNA, which can in turn be reintegrated into the genome for stable expression, in human cells [27]. In this study, we used multiplexed CRISPR/Cas9 to target TSGs in mouse liver and generated mouse models of liver cancer. During targeted TSG disruption in mouse liver cells, extrachromosomal fragments could arise to form small eccDNA either with or without targeted TSG mutations in the transfected founder cells. Small eccDNA with intact TSG target sites could be targeted again by CRISPR/Cas9 to generate targeted mutations if CRISPR/Cas9 remains active in early stage of mouse liver cancer development. Small eccDNA carrying targeted TSG mutations or no targeted mutations could be reintegrated into the genome of proliferating cells by NHEJ or HDR and induce CNVs in daughter cancer cells, promoting IGH [7].

In addition, it appears that transient expression of multiplexed CRISPR/Cas9 is sufficient to initiate persistent oscillation of allelic TSG mutation types and frequencies in single-cell clones as well as strong genomic instability, as a single DSB can induce iterations of chromothripsis in tumor cells [62]. This indicates that persistent generation of small eccDNA in CRISPR/Cas9-induced liver cancer may be assisted by the strong genomic instability manifested by highly activated endogenous DNA damage response, micronuclei formation, increased chromosomal aberrations and hyperploidization in tumor cells. Given the role of DSBs in tumorigenesis, it is also possible that the sheer number of 35 sgRNAs along with *Sp*Cas9 rather than combined TSG mutations play a part in initiation of primary liver tumor in mice. However, a recent study has indicated that DSB induction by controlled expression of the restriction enzyme SacI in the mouse liver induces features of tissue aging in 2 months post DNA double-strand breakage, but no tumors was reported [64]. SacI targets ∼130,000 sites accessible for cleavage in the context of chromatin in the mouse genome, generating a significant level of DSBs [64]. Therefore, it is likely that DSBs induced by CRISPR/Cas9 with 35 sgRNAs alone may not be sufficient to initiate tumors in mouse livers.

Derived from gDNA naturally, eccDNAs range in size from a few hundred bases to megabases, but majority of eccDNAs are smaller than 1 kb [36–40]. In this study, we have identified a few small eccDNAs with or without targeted TSG mutations from single-cell clones of mouse liver cancer model induced by multiplexed CRISPR/Cas9. The size of these small eccDNAs is largely around 400 bp and could not carry a full TSG gene but only TSG fragments with targeted mutations or no targeted mutations. These TSG fragments on their own may not function but exert their effect upon integration into the chromosomes. For example, the TSG fragments on small eccDNAs could replace allelic region of TSGs in the chromosomes of some cells by HDR and alter mutation types in these cells. Thus, the types of targeted mutations and the frequencies of each TSG mutation type oscillate during proliferation of single-cell clones. In some cases, if small eccDNAs harboring targeted TSG mutations are present, active WT TSGs in tumor cells could be inactivated by HDR with these eccDNAs as a template during the development of IGH. Additionally, TSG fragments carrying targeted mutations on eccDNA could be randomly inserted into the chromosomes by NHEJ, not only destabilizing the genome but also potentially initiating a mutational cascade for TSG mutations and CNVs. Therefore, like any other small eccDNAs, small eccDNAs carrying TSGs or TSG fragments with or without mutations could not only be generated but also serve as a fuel for continuing genetic variations in chromosomal TSGs during proliferation of cancer cells, driving IGH and tumor evolution in mouse models of liver cancer induced by multiplexed CRISPR/Cas9. This also suggests a possibility that small eccDNAs carrying TSGs or TSG mutations may be among the sources for the development of IGH in human cancer.

As oncogene-containing eccDNAs promote drug resistance of tumor cells [29–32, 65], this prompted us to test whether small eccDNAs harboring targeted TSG mutations affect the development of drug resistance in CRISPR/Cas9-induced mouse liver cancer. However, after treating mouse liver tumor cells that contain eccDNAs carrying targeted TSG mutations with several drugs including oxaliplatin, olaparib, and sorafenib, we have not yet been able to identify any definitive relationship between drug treatment and oscillation of targeted TSG mutations (data not shown). It is possible that disruption of multiple signaling pathways in these tumor cells may limit the effect of small eccDNAs with targeted TSG mutations on the sensitivity to drug and the development of drug resistance. This application could be improved by further reduction of targeted TSG number in multiplexed CRISPR/Cas9-induced mouse liver cancer. Nevertheless, this study generates a mouse model that can be used to analyze IGH in liver cancer by tracking targeted TSG mutations and to explore the roles of eccDNAs harboring targeted TSG mutations in tumor evolution. In particular, single-cell clones derived from this mouse model of live cancer carry mutations at up to 33 TSGs and could allow efficient immunocompetent allograft, thus providing liver tumor cell lines for studying the interaction between aggressive cancer cells and immune microenvironment.

## Conclusions

By analyzing target site mutations of TSGs in CRISPR/Cas9-induced primary mouse liver tumors and single-cell clones derived from tumor nodules, we found more than two types of allelic variations with varied frequencies at many of TSG target sites within a single-cell clone and significant oscillation of target site mutations in type and frequency at some of TSG target sites in subclones, during proliferation of a subclone, and in mouse subcutaneous graft of a subclone. This study also revealed that genomic instability and generation of small eccDNAs might contribute to this oscillation of target site mutations during cell division. Together, this study is the first to have identified small eccDNAs carrying target site mutations of TSGs as a potential source for the development of IGH in mouse liver cancer induced by multiplexed CRISPR/Cas9. Thus, the relevance to human cancer, particularly human liver cancer, may thus warrant further investigation. 

### Supplementary Information

**Additional file 1: Table S1.** List of target locations with sgRNA sequences, primers for targeted PCR of 34 TSGs and Setd5, and outward primers for circularization junction of eccDNA. **Table S2.** Types, reads and frequencies of target site mutations in CRISPR/Cas9-induced mouse liver tumor tissues. **Table S3.** Types, reads and frequencies of target site mutations in single-cell clones derived from CRISPR/Cas9-induced mouse liver tumor tissues. **Table S4.** Types, reads and frequencies of target site mutations in parental single-cell clones and their subclones. **Table S5.** Types, reads and frequencies of target site mutations in single-cell clones at different time points of proliferation. **Table S6.** Types, reads and frequencies of target site mutations in 4 subcutaneous grafts and 2 single-cell clones derived from each of them. **Table S7.** Identification of eccDNAs from NIH-3T3, 1C3-1 and 6C7 by Circle-Seq. **Table S8.** Comparison of target site mutation types, reads and frequencies between gDNA and eccDNA.

**Additional file 2: Figure S1.** Chromosomal locations of 34 targeted TSGs and the negative control target Setd5 in mouse genome. **Figure S2.** Screening for single sgRNAs targeting 34 TSGs. **Figure S3.** The information on CRISPR/Cas9-induced tumor nodules and single-cell clones derived from tumor modules in mice. **Figure S4.** Representative target mutation oscillation of Rb1, Lkb1, Arid1a and Smad4 between parental clone 6C7 and its subclones. **Figure S5.** No stable expression of SpCas9 detectable in single-cell clones derived from CRISPR/Cas9-induced mouse primary liver tumors. **Figure S6.** Oscillation of target site mutations during proliferation of single-cell subclone 1C3-1, 1C3-2, 1C3-3 and 1C3-4 derived from 1C3. **Figure S7.** Purification of eccDNAs from 1C3-1 and 6C7. **Figure S8.** Identification and mapping of eccDNAs by Circle-Seq in 1C3-1 and 6C7. **Figure S9.** Analysis of Apob-2119 eccDNA and its circularization junction. **Figure S10.** Detection of eccDNA containing target site mutations in single-cell clones. **Figure S11.** Circular barplot for target site mutations of 35 targets in gDNA and eccDNA in representative single-cell clone 1C3-2, 1C3-3, 6C7-2, 6C7-4. **Figure S12.** Identification of circle junction for target eccDNA.

## Data Availability

Microscope datasets for immunochemistry, immunofluorescence, and metaphase analysis and micronuclear staining and the raw datasets for Western blots, agarose gel analysis, and chromatograms of Sanger sequencing are available at Zenodo (https://doi.org/10.5281/zenodo.8262921) [66]. Raw sequencing reads are available from NCBI SRA PRJNA1007250 (https://www.ncbi.nlm.nih.gov/bioproject/PRJNA1007250) for tumor tissues and cell lines [67].

## Abbreviations

AFP: Alpha-fetoprotein
BFB: Breakage-fusion-bridge
BsdR: Blasticidin S deaminase
CK19: Cytokeratin 19
CNV: Copy number variation
CRISPR: Clustered Regularly Interspaced Short Palindromic Repeat
DAPI: 4′,6-Diamidino-2-phenylindole
DSB: DNA double-strand break
eccDNA: Extrachromosomal circular DNA
FBS: Fetal bovine serum
FFPE: Formalin-fixed paraffin-embedded
GAPDH: Glyceraldehyde-3-phosphate dehydrogenase
gDNA: Genomic DNA
GFP: Green fluorescent protein
GP73: Golgi glycoprotein 73
HCC: Hepatocellular carcinoma
HDR: Homology-directed repair
HE: Hematoxylin and eosin
ICC: Intrahepatic cholangiocarcinoma
IGH: Intratumor genetic heterogeneity
ITH: Intratumor heterogeneity
NGS: Next generation sequencing
NHEJ: Non-homologous end joining
ODN: Oligodeoxynucleotide
PBS: Phosphate-buffered saline
SCID: Severe combined immunodeficient
SpCas9: Streptococcus pyogenes Cas9
TSG: Tumor suppressor gene
WT: Wild-type
GFP: Green fluorescent protein
