# Supplementary material for: Small extrachromosomal circular DNA harboring targeted tumor suppressor gene mutations supports intratumor heterogeneity in mouse liver cancer induced by multiplexed CRISPR/Cas9
Source: Genome Med. 2023 Oct 6;15:80. doi: 10.1186/s13073-023-01230-2 (PMC10557318; doi:10.1186/s13073-023-01230-2)
Supplement: Supplementary file 1 — Additional file 1: Table S1. List of target locations with sgRNA sequences, primers for targeted PCR of 34 TSGs and Setd5, and outward primers for circularization junction of eccDNA. Table S2. Types, reads and frequencies of target site mutations in CRISPR/Cas9-induced mouse liver tumor tissues. Table S3. Types, reads and frequencies of target site mutations in single-cell clones derived from CRISPR/Cas9-induced mouse liver tumor tissues. Table S4. Types, reads and frequencies of target site mutations in parental single-cell clones and their subclones. Table S5. Types, reads and frequencies of target site mutations in single-cell clones at different time points of proliferation. Table S6. Types, reads and frequencies of target site mutations in 4 subcutaneous grafts and 2 single-cell clones derived from each of them. Table S7. Identification of eccDNAs from NIH-3T3, 1C3-1 and 6C7 by Circle-Seq. Table S8. Comparison of target site mutation types, reads and frequencies between gDNA and eccDNA. [file 13073_2023_1230_MOESM1_ESM.zip › Supplementary Information Additional file 1_082023.pdf]

Supplementary Information

Additional file 1: Supplementary tables

Small extrachromosomal circular DNA harboring targeted tumor suppressor gene mutations supports intratumor heterogeneity in mouse liver cancer induced by multiplexed CRISPR/Cas9

Tao Guo[1,2,†], Guo-Qiao Chen[1,2,†], Xu-Fan Li[1,2,†], Meng Wang[1,2], Kun-Ming Liu[1,2], Xiao-Ying Yang[1,2], Si-Cheng Liu[1,2], Yi-Li Feng[1,2], Peng-Yuan Liu[1,2,*], Hui Lin[1,*], and An-Yong Xie[1,2,*]

[1] Innovation Center for Minimally Invasive Technique and Device, Department of General Surgery, Sir Run Run Shaw Hospital, Zhejiang University School of Medicine, Hangzhou, Zhejiang 310019, P. R. China

[2] Institute of Translational Medicine, Zhejiang University School of Medicine and Zhejiang University Cancer Center, Hangzhou, Zhejiang 310029, P. R. China

[*] Corresponding authors:

An-Yong Xie, Ph.D., Institute of Translational Medicine, Zhejiang University School of Medicine, 268 Kai Xuan Rd, Hangzhou, Zhejiang 310029, China. Tel: +86 0571 86971680; Fax: +86 0571 88981576; Email: anyongxie@zju.edu.cn (lead contact).

Hui Lin, M.D., Department of General Surgery, Sir Run Run Shaw Hospital, Zhejiang University School of Medicine, 3 East Qingchun Rd, Hangzhou, Zhejiang 310016, China. Email: 369369@zju.edu.cn.

Peng-Yuan Liu, Ph.D., Institute of Translational Medicine, Zhejiang University School of Medicine, 268 Kai Xuan Rd, Hangzhou, Zhejiang 310029, China. Email: pyliu@zju.edu.cn.

[†] These authors contributed equally to this work

Additional file 1: Supplementary tables

Table S1: List of target locations with sgRNA sequences, primers for targeted PCR of 34 TSGs and *Setd5*, and outward primers for circularization junction of eccDNA.

Table S2: Types, reads and frequencies of target site mutations in CRISPR/Cas9-induced mouse liver tumor tissues.

Table S3: Types, reads and frequencies of target site mutations in single-cell clones derived from CRISPR/Cas9-induced mouse liver tumor tissues.

Table S4: Types, reads and frequencies of target site mutations in parental single-cell clones and their subclones. 1C3-1, -2, -3 and -4: subclones of 1C3; 6C7-1, -2, -3, -4, -5, -6, -7 and -8: subclones of 6C7.

Table S5: Types, reads and frequencies of target site mutations in single-cell clones at different time points of proliferation.

Table S6: Types, reads and frequencies of target site mutations in 4 subcutaneous grafts and 2 single-cell clones derived from each of them.

Table S7: Identification of eccDNAs from NIH-3T3, 1C3-1 and 6C7 by Circle-Seq.

Table S8: Comparison of target site mutation types, reads and frequencies between gDNA and eccDNA.
