# Supplementary material for: Small extrachromosomal circular DNA harboring targeted tumor suppressor gene mutations supports intratumor heterogeneity in mouse liver cancer induced by multiplexed CRISPR/Cas9
Source: Genome Med. 2023 Oct 6;15:80. doi: 10.1186/s13073-023-01230-2 (PMC10557318; doi:10.1186/s13073-023-01230-2)
Supplement: Supplementary file 2 — Additional file 2: Figure S1. Chromosomal locations of 34 targeted TSGs and the negative control target Setd5 in mouse genome. Figure S2. Screening for single sgRNAs targeting 34 TSGs. Figure S3. The information on CRISPR/Cas9-induced tumor nodules and single-cell clones derived from tumor modules in mice. Figure S4. Representative target mutation oscillation of Rb1, Lkb1, Arid1a and Smad4 between parental clone 6C7 and its subclones. Figure S5. No stable expression of SpCas9 detectable in single-cell clones derived from CRISPR/Cas9-induced mouse primary liver tumors. Figure S6. Oscillation of target site mutations during proliferation of single-cell subclone 1C3-1, 1C3-2, 1C3-3 and 1C3-4 derived from 1C3. Figure S7. Purification of eccDNAs from 1C3-1 and 6C7. Figure S8. Identification and mapping of eccDNAs by Circle-Seq in 1C3-1 and 6C7. Figure S9. Analysis of Apob-2119 eccDNA and its circularization junction. Figure S10. Detection of eccDNA containing target site mutations in single-cell clones. Figure S11. Circular barplot for target site mutations of 35 targets in gDNA and eccDNA in representative single-cell clone 1C3-2, 1C3-3, 6C7-2, 6C7-4. Figure S12. Identification of circle junction for target eccDNA. [file 13073_2023_1230_MOESM2_ESM.pdf]

Supplementary Information

Additional file 2: Supplementary figures

Small extrachromosomal circular DNA harboring targeted tumor suppressor gene mutations supports intratumor heterogeneity in mouse liver cancer induced by multiplexed CRISPR/Cas9

Tao Guo[1,2,†], Guo-Qiao Chen[1,2,†], Xu-Fan Li[1,2,†], Meng Wang[1,2], Kun-Ming Liu[1,2], Xiao-Ying Yang[1,2], Si-Cheng Liu[1,2], Yi-Li Feng[1,2], Peng-Yuan Liu[1,2,*], Hui Lin[1,*], and An-Yong Xie[1,2,*]

[1] Innovation Center for Minimally Invasive Technique and Device, Department of General Surgery, Sir Run Run Shaw Hospital, Zhejiang University School of Medicine, Hangzhou, Zhejiang 310019, P. R. China

[2] Institute of Translational Medicine, Zhejiang University School of Medicine and Zhejiang University Cancer Center, Hangzhou, Zhejiang 310029, P. R. China

[*] Corresponding authors:

An-Yong Xie, Ph.D., Institute of Translational Medicine, Zhejiang University School of Medicine, 268 Kai Xuan Rd, Hangzhou, Zhejiang 310029, China. Tel: +86 0571 86971680; Fax: +86 0571 88981576; Email: anyongxie@zju.edu.cn (lead contact).

Hui Lin, M.D., Department of General Surgery, Sir Run Run Shaw Hospital, Zhejiang University School of Medicine, 3 East Qingchun Rd, Hangzhou, Zhejiang 310016, China. Email: 369369@zju.edu.cn.

Peng-Yuan Liu, Ph.D., Institute of Translational Medicine, Zhejiang University School of Medicine, 268 Kai Xuan Rd, Hangzhou, Zhejiang 310029, China. Email: pyliu@zju.edu.cn.

[†] These authors contributed equally to this work

Additional file 2: Supplement figures

Figure S1. Chromosomal locations of 34 targeted TSGs and the negative control target *Setd5* in mouse genome. Locations of TSGs and *Setd5* are indicated on mouse chromosomes. Color denotes cancer signaling pathways and TSGs associated.

Figure S2. Screening for single sgRNAs targeting 34 TSGs.

(**A**) Schematic of the gene editing reporter. The reporter was modified from the BGN reporter previously established and contains a *BsdR-GFP* fusion gene in which GFP is out of frame. BsdR encodes blasticidin S deaminase. The I-SceI and EcoRI sites in the *BsdR* portion are designed for insertion of target sequence used to test the efficiency of Cas9-sgRNA. After transfection of mammalian cells, expression of Cas9-sgRNA induces a site-specific DSB at its target in the reporter, and repair of this DSB by mutagenic NHEJ could reframe GFP, generating in-frame GFP and making cells GFP^+. Thus, the frequency of Cas9-induced GFP^+ cells reflects the efficiency of sgRNA.

(**B**) Percentages of Cas9-induced GFP^+ cells with 3 different sgRNAs for each TSG target.

Figure S3. The information on CRISPR/Cas9-induced tumor nodules and single-cell clones derived from tumor modules in mice. Dosage per sgRNA used in 34-sgRNA library and days for tumor induction are indicated.

Figure S4. Representative target mutation oscillation of *Rb1* (A), *Lkb1* (B), *Arid1a* (C) and *Smad4* (D) between parental clone 6C7 and its subclones.

Figure S5. No stable expression of SpCas9 detectable in single-cell clones derived from CRISPR/Cas9-induced mouse primary liver tumors.

(**A**) Western blot of Flag-tagged *Sp*Cas9 proteins stably expressed in single-cell clones derived from CRISPR/Cas9-induced mouse primary liver tumors. Flag-tagged *Sp*Cas9 proteins in the control NIH3T3 cells transiently transfected with expression plasmids was used as a positive control.

(**B,C**) Detection of stable *Sp*Cas9 activity in single-cell clones 1C3-1 (**B**) and several other single-cell clones (**C**) by deep sequencing. Single-cell clones were transfected with the expression plasmid for *Col1a1* sgRNA or *Rosa26* sgRNA together with (+)

or without (–) the *Sp*Cas9 expression plasmid. gDNA was isolated from cells at 72 h post transfection and the editing frequency was determined by targeted PCR amplicon deep sequencing.

Figure S6. Oscillation of target site mutations during proliferation of single-cell subclone 1C3-1, 1C3-2, 1C3-3 and 1C3-4 derived from 1C3. Changes in target site mutation profiles of 1C3-1, 1C3-2, 1C3-3 and 1C3-4 at indicated time points of cell proliferation (i.e., Day 0, 15 and 30). Gene targets and their mutation types are shown on top and at bottom, respectively. The numbers flanking Del or Ins indicated at bottom are the numbers of deleted or inserted nucleotides. √Frequency representing square root of mutation frequencies is indicated from the highest 1 in dark green to the lowest 0 in white. The top line chart and the boxplot under show the number of single-cell clones with a specific mutation type, i.e., Number of clones, and the percentage distribution of a specific mutation type in all single-cell clones, i.e., Proportion (%), respectively.

Figure S7. Purification of eccDNAs from 1C3-1 and 6C7.
(**A**) Experimental outline of eccDNA purification and identification by Circle-Seq.
(**B**) Validation of linear gDNA removal from eccDNAs by targeted PCR amplification of *Actb* in cell lines indicated. After gDNA was isolated, Plasmid-Safe ATP-Dependent DNase was added to digest linear gDNA and eccDNA was subsequently purified. gDNA and eccDNA purified for PCR were indicated by "–" and "+" DNase, respectively. PCR products were stained with ethidium bromide after DNA gel electrophoresis. M: DNA marker.
(**C,D**) Removal of linear gDNA and mitochondrial DNA from eccDNAs validated by targeted PCR amplification of the nuclear gene *Cox5b* (**C**) and the mitochondrial gene *mt-Co1* (**D**) in the control NIH-3T3 cells, 6C7 and 1C3-1 cells as indicated. After crude eccDNAs were isolated by column chromatography, PacI and Plasmid-Safe ATP-Dependent DNase were added to digest mitochondrial DNA and linear gDNA, respectively, allowing further purification of eccDNAs. PCR products of either gDNA, crude eccDNA or eccDNA indicated for nuclear gene *Cox5b* and mitochondrial gene *mt-Co1* were stained with SYBR Gold stain after DNA gel electrophoresis. M: DNA marker.

Figure S8. Identification and mapping of eccDNAs by Circle-Seq in 1C3-1 and 6C7.

(**A**) Chromosomal distribution of eccDNAs from NIH-3T3, 1C3-1 and 6C7 cells. The target gene sites by 35 sgRNAs for 34 TSGs and *Setd6* are depicted in red lines on 21 chromosomes. Mapping of eccDNAs from each cell line to 21 chromosomes is also indicated.

(**B-D**) Identification of eccDNAs carrying genes targeted by 35 sgRNAs in NIH-3T3 (**B**), 1C3-1 (**C**) and 6C7 cells (**D**). The overlap of gene regions between eccDNAs and genes targeted by 35 sgRNAs were determined by Venn diagrams.

Figure S9. Analysis of *Apob*-2119 eccDNA and its circularization junction.

(A) Schematic for analysis of *Apob*-2119 eccDNA and its circularization junction by Sanger sequencing. PCR amplification of *Apob*-2119 eccDNA and its circularization junction was performed with two pairs of outward primers (i.e., F1/R1 and F1/R2) and one pair of inward primers (i.e., F2/R2) as indicated, respectively. PCR products were cloned into the pUC19 vector for Sanger sequencing.

(**B**) PCR products of *Apob*-2119 eccDNA from NIH-3T3, 1C3-1 and 6C7 were analyzed by DNA gel electrophoresis. M: DNA marker.

Figure S10. Detection of eccDNA containing target site mutations in single-cell clones.

(**A**) Experimental outline of eccDNA purification and detection. gDNA was extracted from single-cell clones and digested with Plasmid-Safe ATP-Dependent DNase to enrich eccDNA. Targeted PCR amplicon deep sequencing was performed on gDNA and eccDNA respectively to identify target site mutations in gDNA or eccDNA.

(**B**) Read counts of 34 TSG targets and the *Setd5* target in eccDNA by targeted PCR amplicon deep sequencing from six single-cell clones.

(**C**) Ratio of total reads of each target in gDNA or in eccDNA to all target reads. After PCR amplification, the equal amounts of PCR products of each locus with two different templates were prepared for deep sequencing. All target reads were counted firstly, ratio of reads of each target to all target reads were calculated.

(**D**) Relative read ratio of eccDNA to gDNA. In order to exhibit clearly, we used relative read ratio of eccDNA to gDNA to describe the change of deep sequencing results those targets that over 1 indicated eccDNA existence.

Figure S11. Circular barplot for target site mutations of 35 targets in gDNA and eccDNA in representative single-cell clone 1C3-2 (A), 1C3-3 (B), 6C7-2 (C), 6C7-4 (D). The column in the inner circle for each target gene were relative read ratios of eccDNA to gDNA for each target site from deep sequencing data. The stacked barplot in the outer circle represents the mutation pattern at indicated target sites in eccDNA and gDNA. Mutation types are shown in color. Total read counts of eccDNA and gDNA are listed. *, invalid read counts at indicated sites, which are defined as <0.1% of total read counts from eccDNA or gDNA.

Figure S12. Identification of circle junction for target eccDNA.
(**A**) Schematic of target eccDNA formation and circularization junction analysis. Extrachromosomal linear DNA fragments were generated and circularized to form eccDNA. PCR amplification of circularization junction was performed with a pair of outward primers and PCR products cloned into the pUC19 vector for Sanger sequencing.
(**B**) Original target sequence for eccDNA formation containing *Atm*, *Rb1* or *Kmt2c* target sites. > and < indicate the site for circularization. Exons are shown in red with PAM in bold. Sequences underlined are outward primer pair for PCR of circularization junctions.

Figure S1

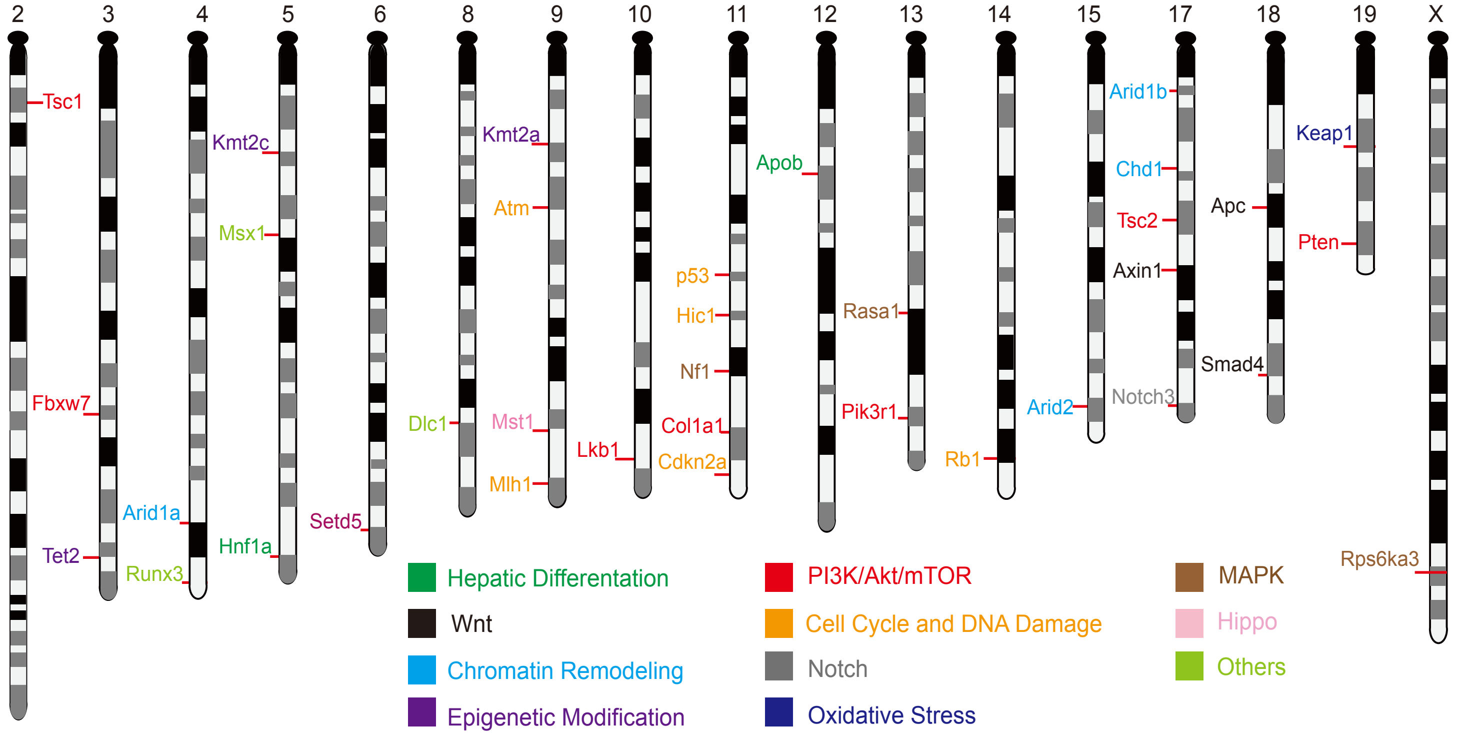

2
3
4
5
6
8
9
10
11
12
13
14
15
17
18
19
X
Tsc1
Fbxw7
Tet2
Runx3
Arid1a
Kmt2c
Msx1
Hnf1a
Setd5
Kmt2a
Atm
Dlc1
Mst1
Mlh1
Lkb1
p53
Hic1
Nf1
Col1a1
Cdkn2a
Apob
Rasa1
Pik3r1
Rb1
Arid1b
Chd1
Tsc2
Axin1
Arid2
Notch3
Apc
Smad4
Keap1
Pten
Rps6ka3
Hepatic Differentation
Wnt
Chromatin Remodeling
Epigenetic Modification
PI3K/Akt/mTOR
Cell Cycle and DNA Damage
Notch
Oxidative Stress
MAPK
Hippo
Others

Figure S2

A

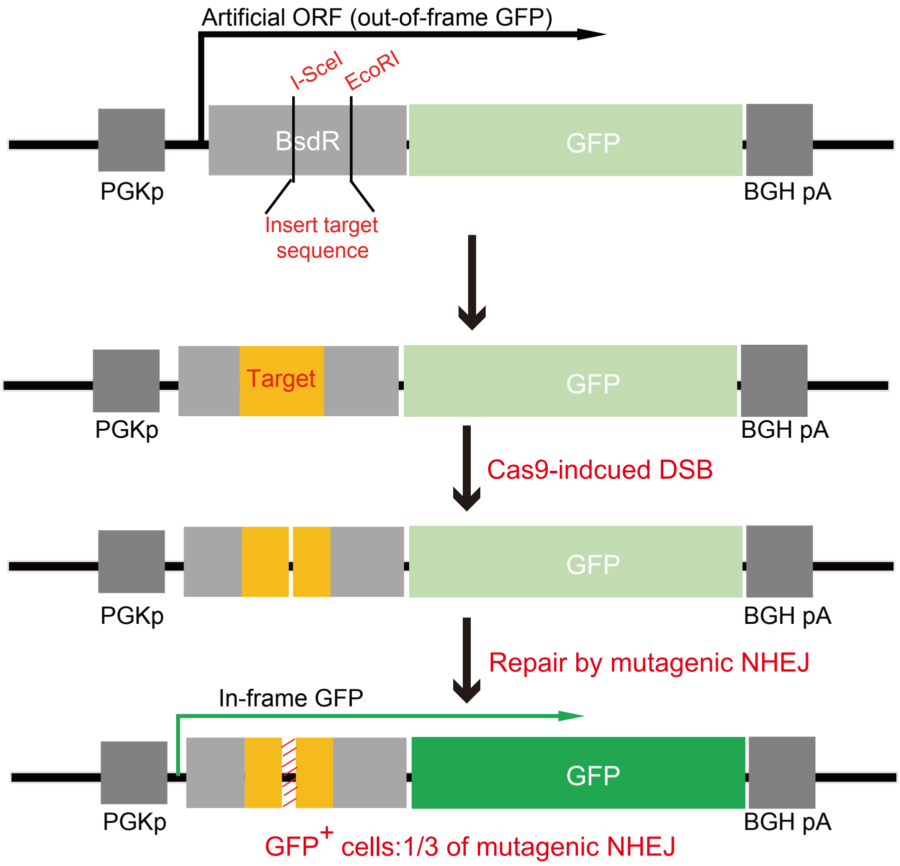
Artificial ORF (out-of-frame GFP)
I-SceI
EcoRI
BsdR
GFP
PGKp
BGH pA
Insert target sequence
Target
Cas9-indcued DSB
Repair by mutagenic NHEJ
In-frame GFP
GFP+ cells:1/3 of mutagenic NHEJ

B

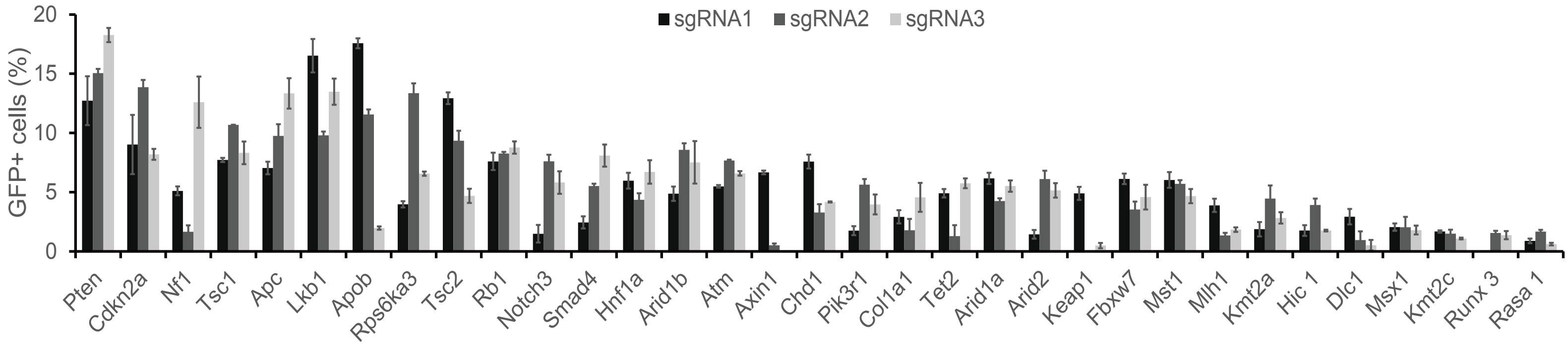
GFP+ cells (%)
20
15
10
5
0
sgRNA1
sgRNA2
sgRNA3
Pten
Cdkn2a
Nf1
Tsc1
Apc
Lkb1
Apob
Rps6ka3
Tsc2
Rb1
Notch3
Smad4
Hnf1a
Arid1b
Atm
Axin1
Chd1
Pik3r1
Col1a1
Tet2
Arid1a
Arid2
Keap1
Fbxw7
Mst1
Mlh1
Kmt2a
Hic 1
Dlc1
Msx1
Kmt2c
Runx 3
Rasa 1

Figure S3

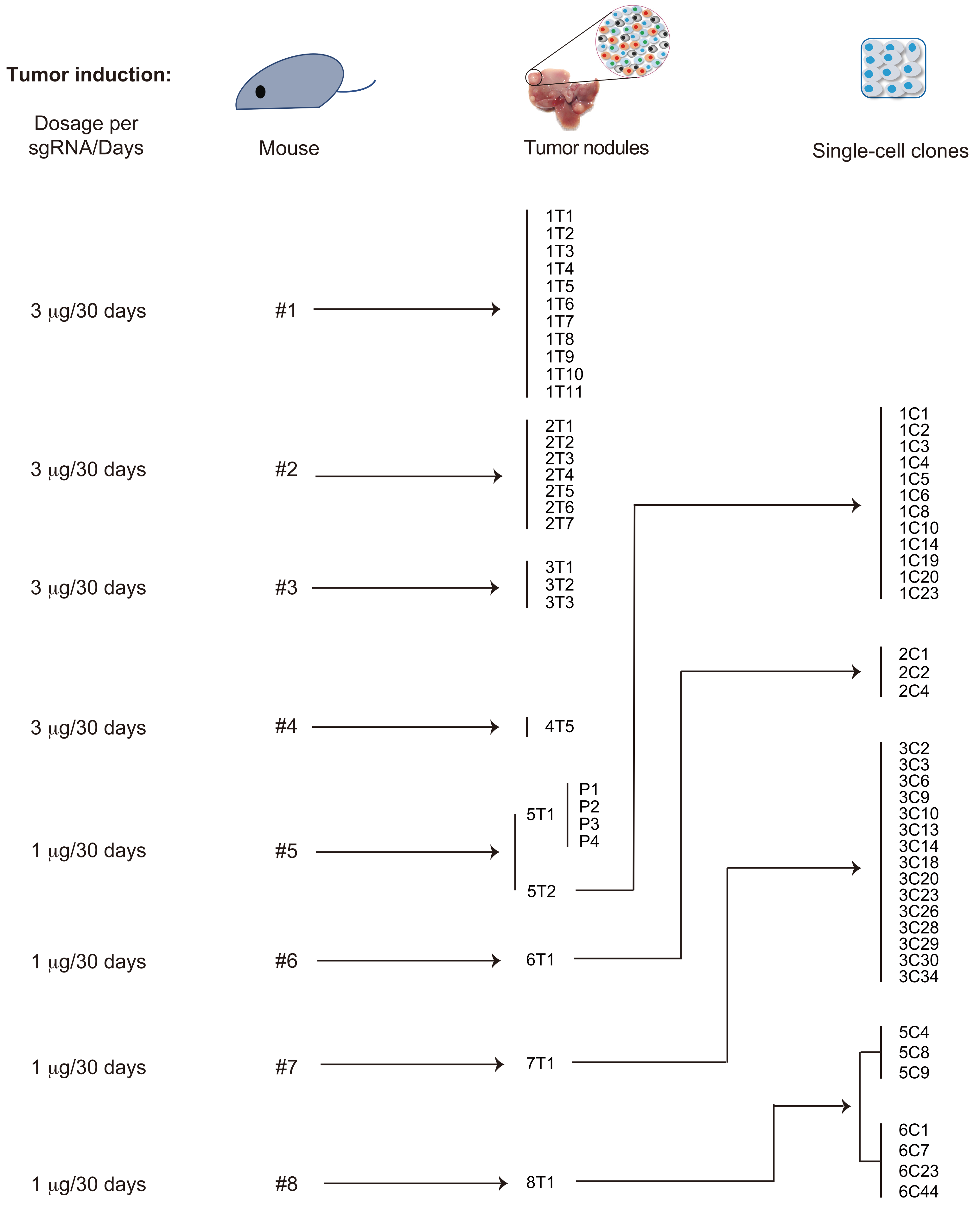

Tumor induction:
Dosage per sgRNA/Days
Mouse
Tumor nodules
Single-cell clones
3 μg/30 days
#1
1T1
1T2
1T3
1T4
1T5
1T6
1T7
1T8
1T9
1T10
1T11
3 μg/30 days
#2
2T1
2T2
2T3
2T4
2T5
2T6
2T7
3 μg/30 days
#3
3T1
3T2
3T3
3 μg/30 days
#4
4T5
1 μg/30 days
#5
5T1
P1
P2
P3
P4
5T2
1 μg/30 days
#6
6T1
1 μg/30 days
#7
7T1
1 μg/30 days
#8
8T1
1C1
1C2
1C3
1C4
1C5
1C6
1C8
1C10
1C14
1C19
1C20
1C23
2C1
2C2
2C4
3C2
3C3
3C6
3C9
3C10
3C13
3C14
3C18
3C20
3C23
3C26
3C28
3C29
3C30
3C34
5C4
5C8
5C9
6C1
6C7
6C23
6C44

Figure S4

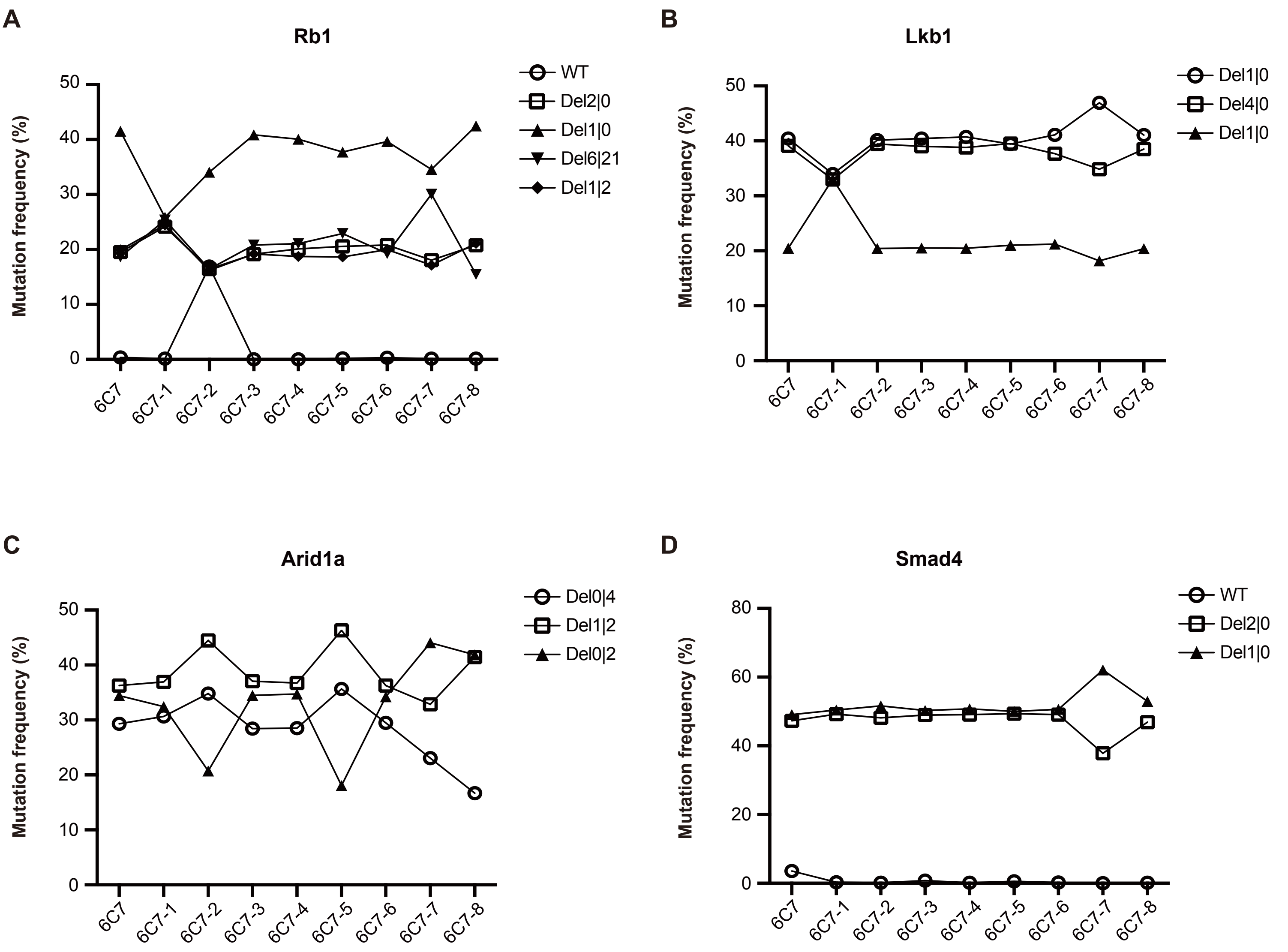
A
Rb1
WT
Del2|0
Del1|0
Del6|21
Del1|2
Mutation frequency (%)
0
10
20
30
40
50
6C7
6C7-1
6C7-2
6C7-3
6C7-4
6C7-5
6C7-6
6C7-7
6C7-8
B
Lkb1
Del1|0
Del4|0
Del1|0
C
Arid1a
Del0|4
Del1|2
Del0|2
D
Smad4
WT
Del2|0
Del1|0
60
80

Figure S5

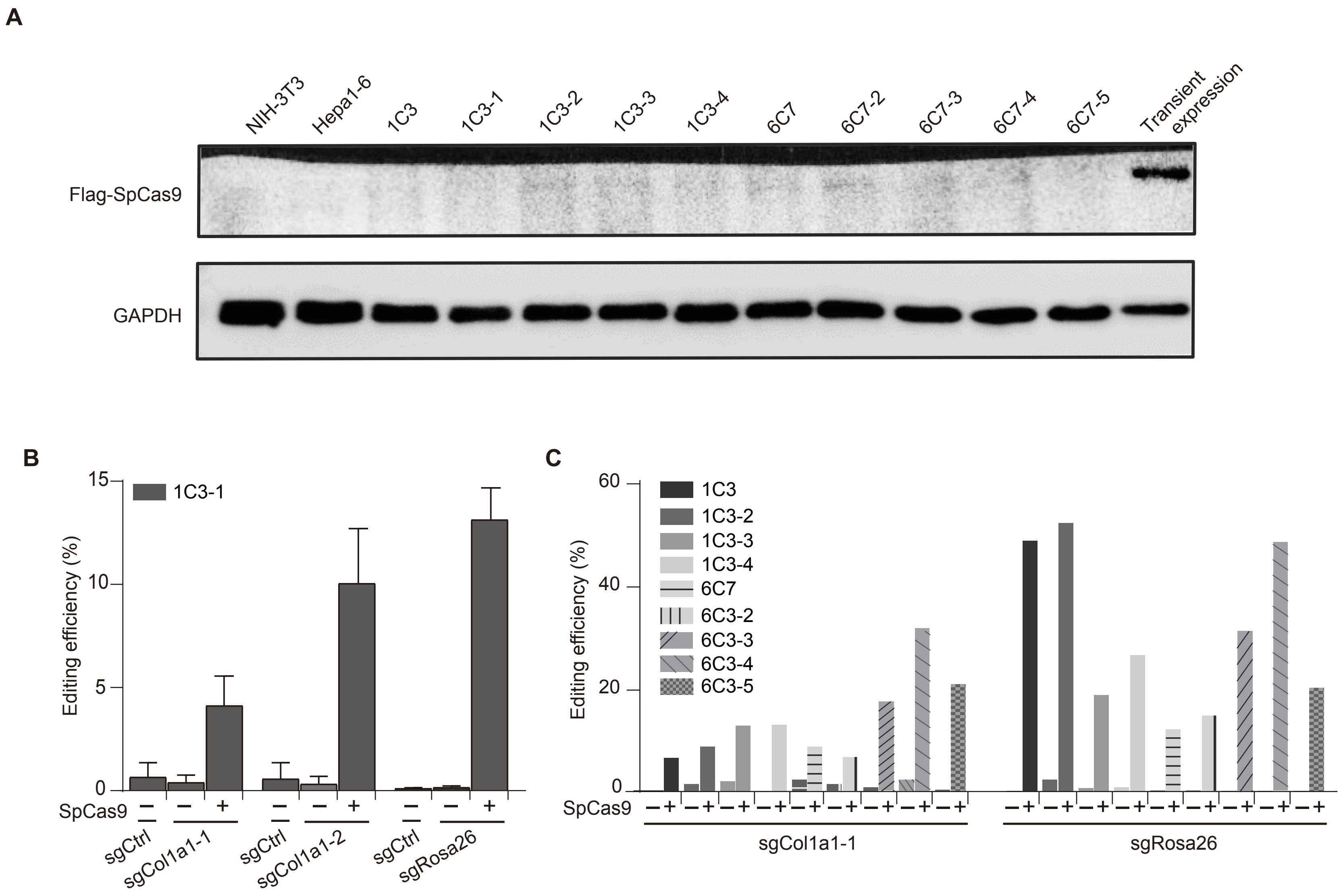
A
NIH-3T3
Hepa1-6
1C3
1C3-1
1C3-2
1C3-3
1C3-4
6C7
6C7-2
6C7-3
6C7-4
6C7-5
Transient expression
Flag-SpCas9
GAPDH
B
1C3-1
Editing efficiency (%)
15
10
5
0
SpCas9
sgCtrl
sgCol1a1-1
sgCol1a1-2
sgRosa26
C
1C3
1C3-2
1C3-3
1C3-4
6C7
6C3-2
6C3-3
6C3-4
6C3-5
Editing efficiency (%)
60
40
20
0
SpCas9
sgCol1a1-1
sgRosa26

Figure S6

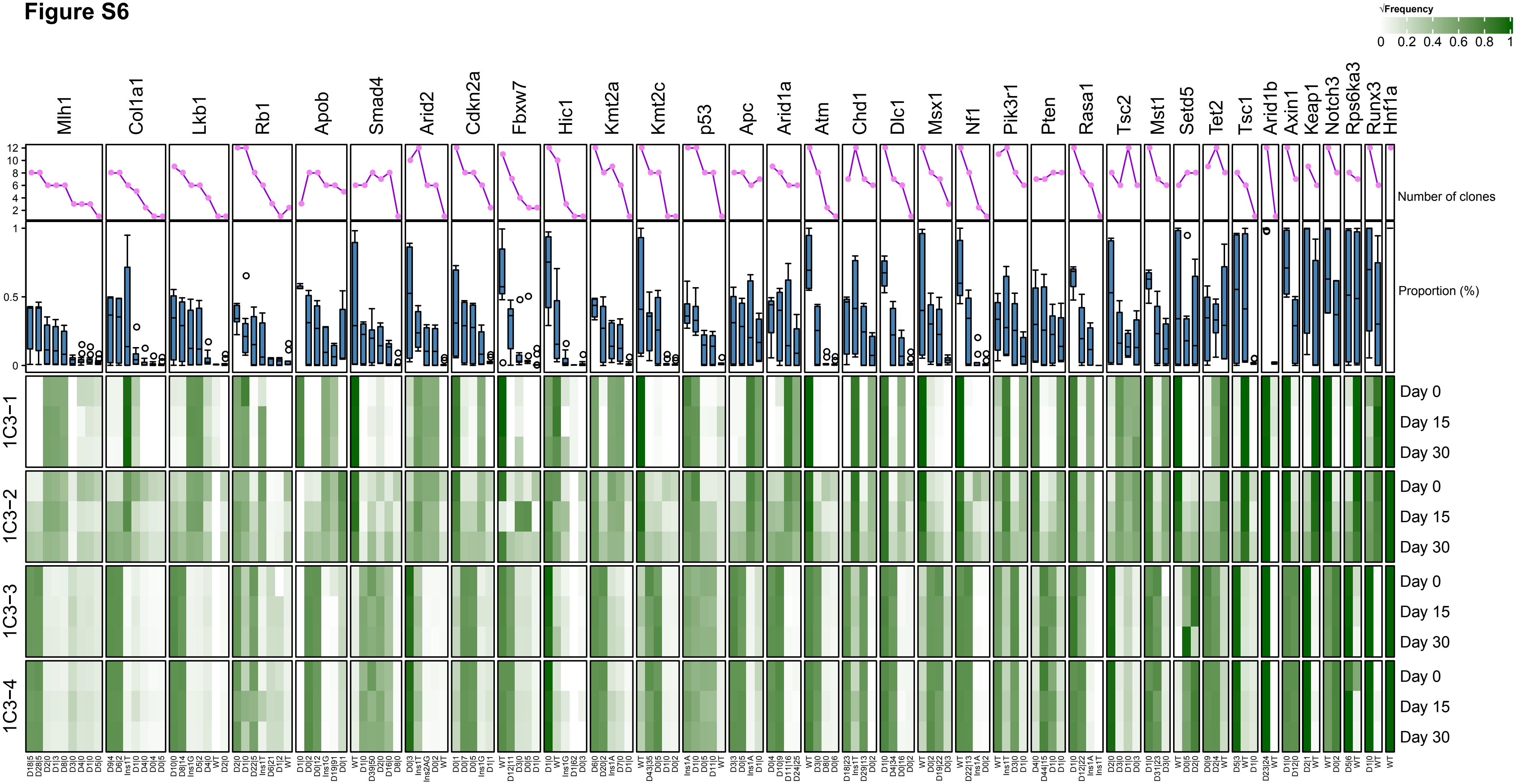
√Frequency
0 0.2 0.4 0.6 0.8 1
Mlh1
Col1a1
Lkb1
Rb1
Apob
Smad4
Arid2
Cdkn2a
Fbxw7
Hic1
Kmt2a
Kmt2c
p53
Apc
Arid1a
Atm
Chd1
Dlc1
Msx1
Nf1
Pik3r1
Pten
Rasa1
Tsc2
Mst1
Setd5
Tet2
Tsc1
Arid1b
Axin1
Keap1
Notch3
Rps6ka3
Runx3
Hnf1a
Number of clones
Proportion (%)
1C3−1
1C3−2
1C3−3
1C3−4
Day 0
Day 15
Day 30

Figure S7

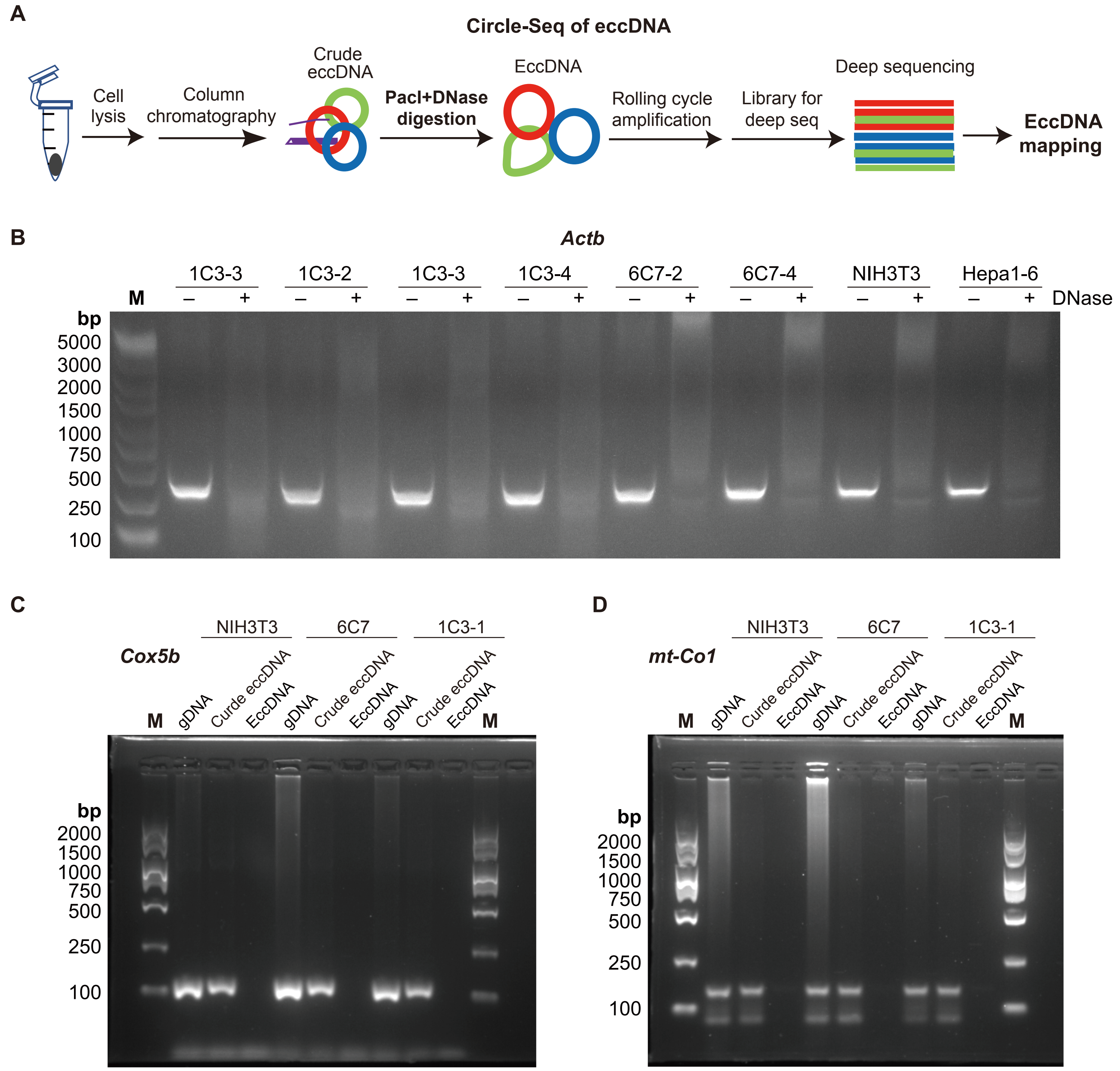
A
Circle-Seq of eccDNA
Cell lysis
Column chromatography
Crude eccDNA
PacI+DNase digestion
EccDNA
Rolling cycle amplification
Library for deep seq
Deep sequencing
EccDNA mapping
B
Actb
1C3-3
1C3-2
1C3-3
1C3-4
6C7-2
6C7-4
NIH3T3
Hepa1-6
M
– + – + – + – + – + – + – + – +
DNase
bp
5000
3000
2000
1500
1000
750
500
250
100
C
Cox5b
NIH3T3
6C7
1C3-1
M
gDNA
Curde eccDNA
EccDNA
gDNA
Crude eccDNA
EccDNA
gDNA
Crude eccDNA
EccDNA
M
bp
2000
1500
1000
750
500
250
100
D
mt-Co1
NIH3T3
6C7
1C3-1
M
gDNA
Crude eccDNA
EccDNA
gDNA
Crude eccDNA
EccDNA
gDNA
Crude eccDNA
EccDNA
M
bp
2000
1500
1000
750
500
250
100

Figure S8

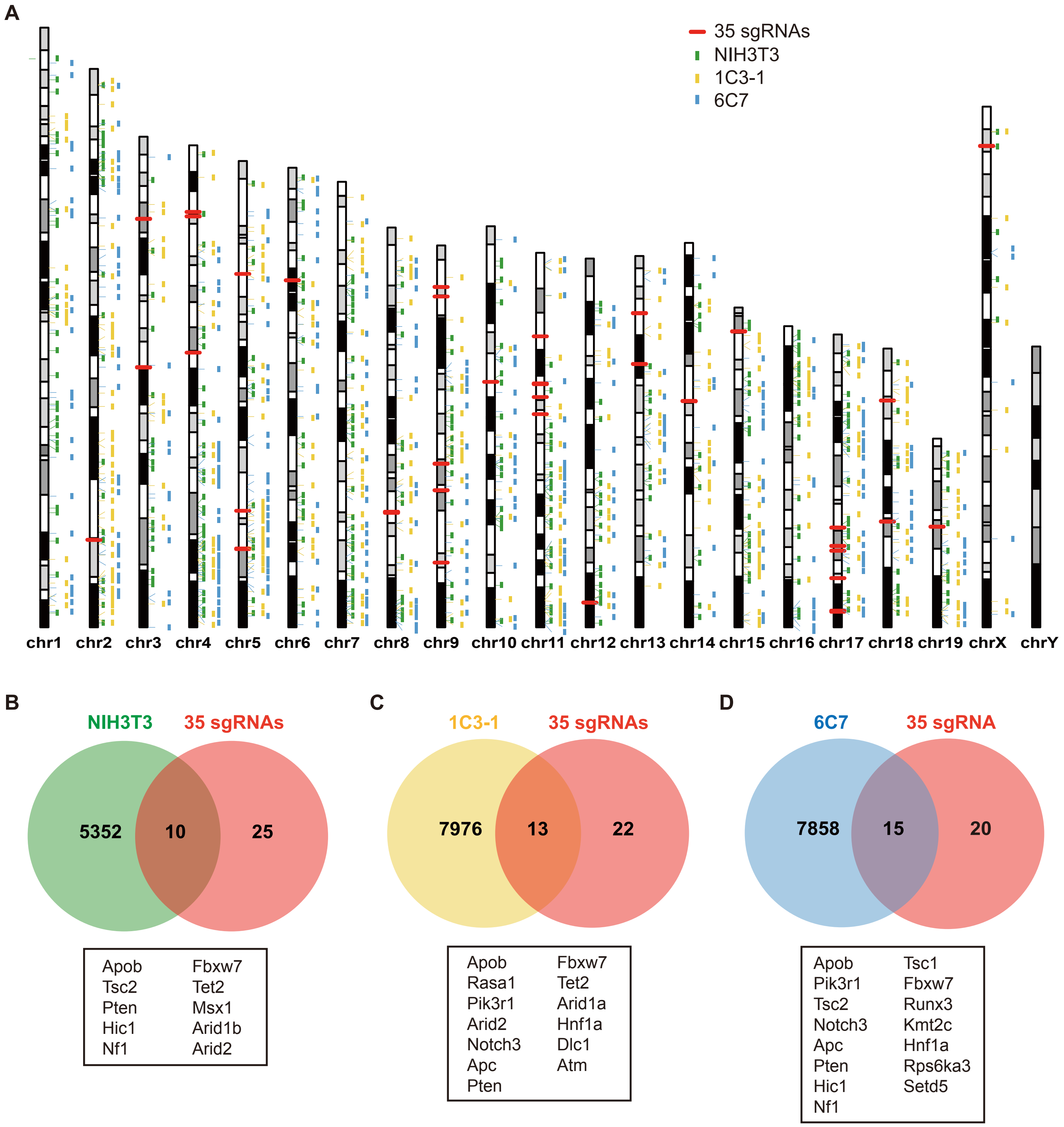
A
35 sgRNAs
NIH3T3
1C3-1
6C7
chr1 chr2 chr3 chr4 chr5 chr6 chr7 chr8 chr9 chr10 chr11 chr12 chr13 chr14 chr15 chr16 chr17 chr18 chr19 chrX chrY
B
NIH3T3
35 sgRNAs
5352
10
25
Apob Tsc2 Pten Hic1 Nf1 Fbxw7 Tet2 Msx1 Arid1b Arid2
C
1C3-1
35 sgRNAs
7976
13
22
Apob Rasa1 Pik3r1 Arid2 Notch3 Apc Pten Fbxw7 Tet2 Arid1a Hnf1a Dlc1 Atm
D
6C7
35 sgRNA
7858
15
20
Apob Pik3r1 Tsc2 Notch3 Apc Pten Hic1 Nf1 Tsc1 Fbxw7 Runx3 Kmt2c Hnf1a Rps6ka3 Setd5

Figure S9

A

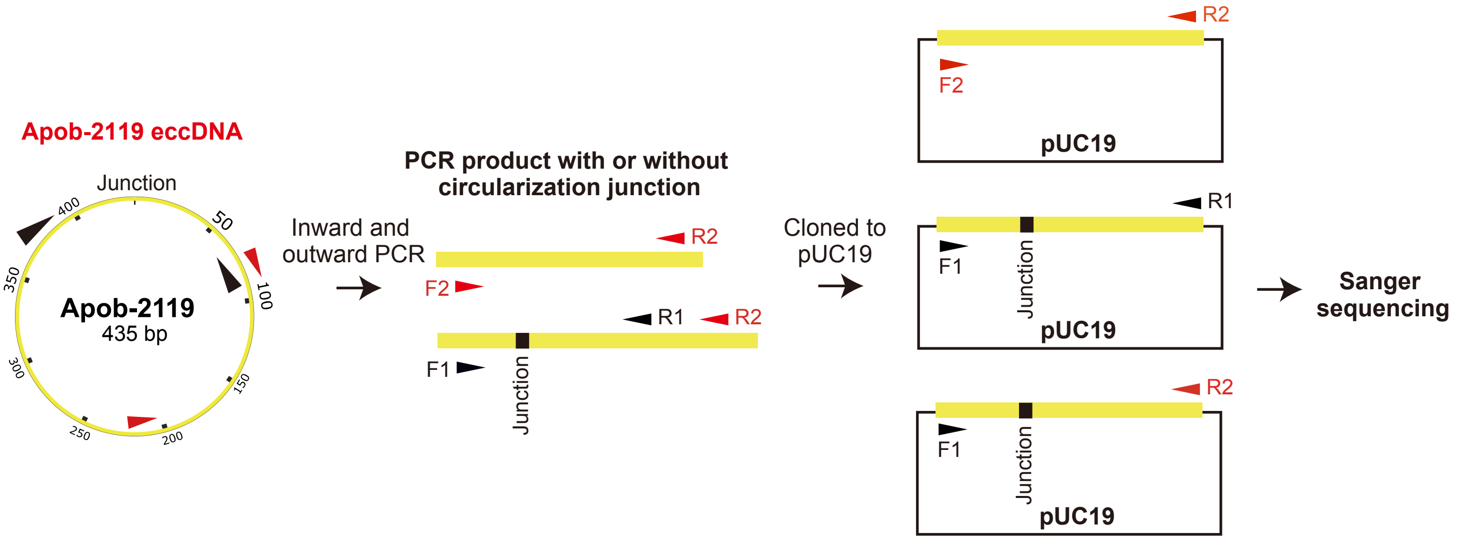
Apob-2119 eccDNA
Junction
Apob-2119
435 bp
400
50
100
150
200
250
300
350
Inward and
outward PCR
PCR product with or without
circularization junction
F2
R2
F1
R1
R2
Junction
Cloned to
pUC19
R2
F2
pUC19
R1
F1
Junction
pUC19
R2
F1
Junction
pUC19
Sanger
sequencing

B

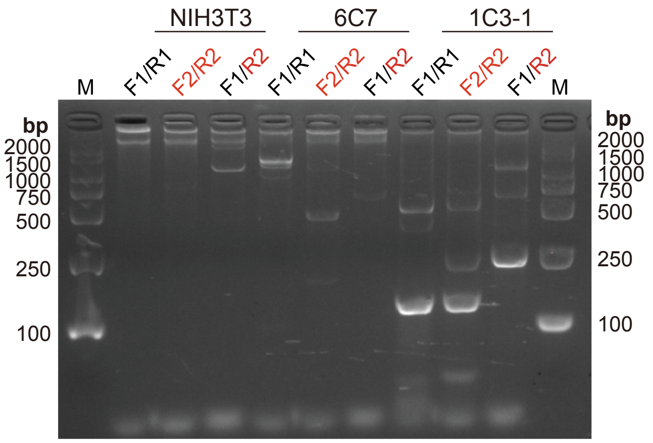
NIH3T3
6C7
1C3-1
M
F1/R1
F2/R2
F1/R2
F1/R1
F2/R2
F1/R2
F1/R1
F2/R2
F1/R2
M
bp
2000
1500
1000
750
500
250
100
bp
2000
1500
1000
750
500
250
100

Figure S10

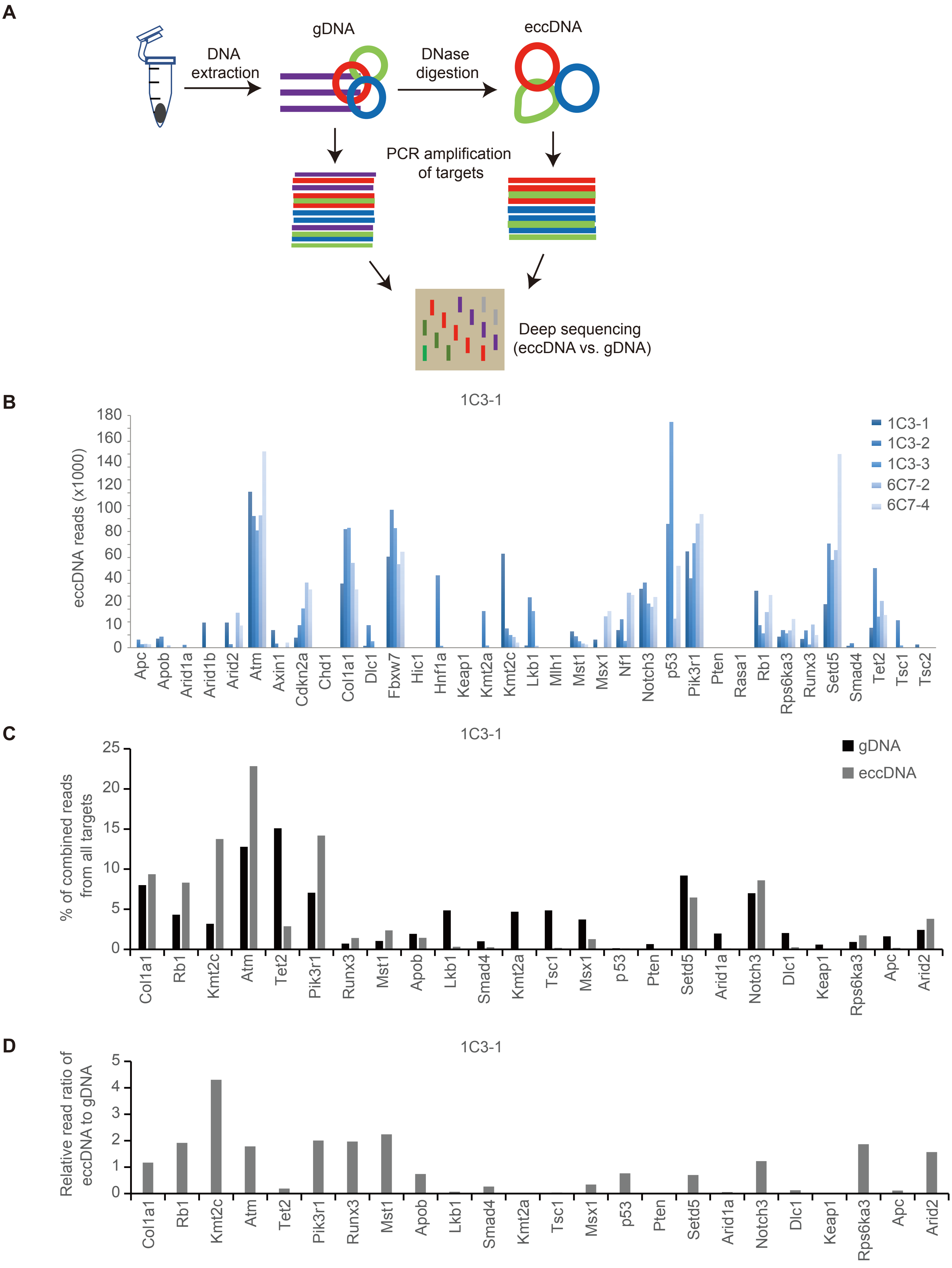
A
DNA extraction
gDNA
DNase digestion
eccDNA
PCR amplification of targets
Deep sequencing (eccDNA vs. gDNA)
B
1C3-1
eccDNA reads (x1000)
1C3-1
1C3-2
1C3-3
6C7-2
6C7-4
Apc Apob Arid1a Arid1b Arid2 Atm Axin1 Cdkn2a Chd1 Col1a1 Dlc1 Fbxw7 Hic1 Hnf1a Keap1 Kmt2a Kmt2c Lkb1 Mlh1 Mst1 Msx1 Nf1 Notch3 p53 Pik3r1 Pten Rasa1 Rb1 Rps6ka3 Runx3 Setd5 Smad4 Tet2 Tsc1 Tsc2
C
1C3-1
% of combined reads from all targets
gDNA
eccDNA
Col1a1 Rb1 Kmt2c Atm Tet2 Pik3r1 Runx3 Mst1 Apob Lkb1 Smad4 Kmt2a Tsc1 Msx1 p53 Pten Setd5 Arid1a Notch3 Dlc1 Keap1 Rps6ka3 Apc Arid2
D
1C3-1
Relative read ratio of eccDNA to gDNA
Col1a1 Rb1 Kmt2c Atm Tet2 Pik3r1 Runx3 Mst1 Apob Lkb1 Smad4 Kmt2a Tsc1 Msx1 p53 Pten Setd5 Arid1a Notch3 Dlc1 Keap1 Rps6ka3 Apc Arid2

Figure S11

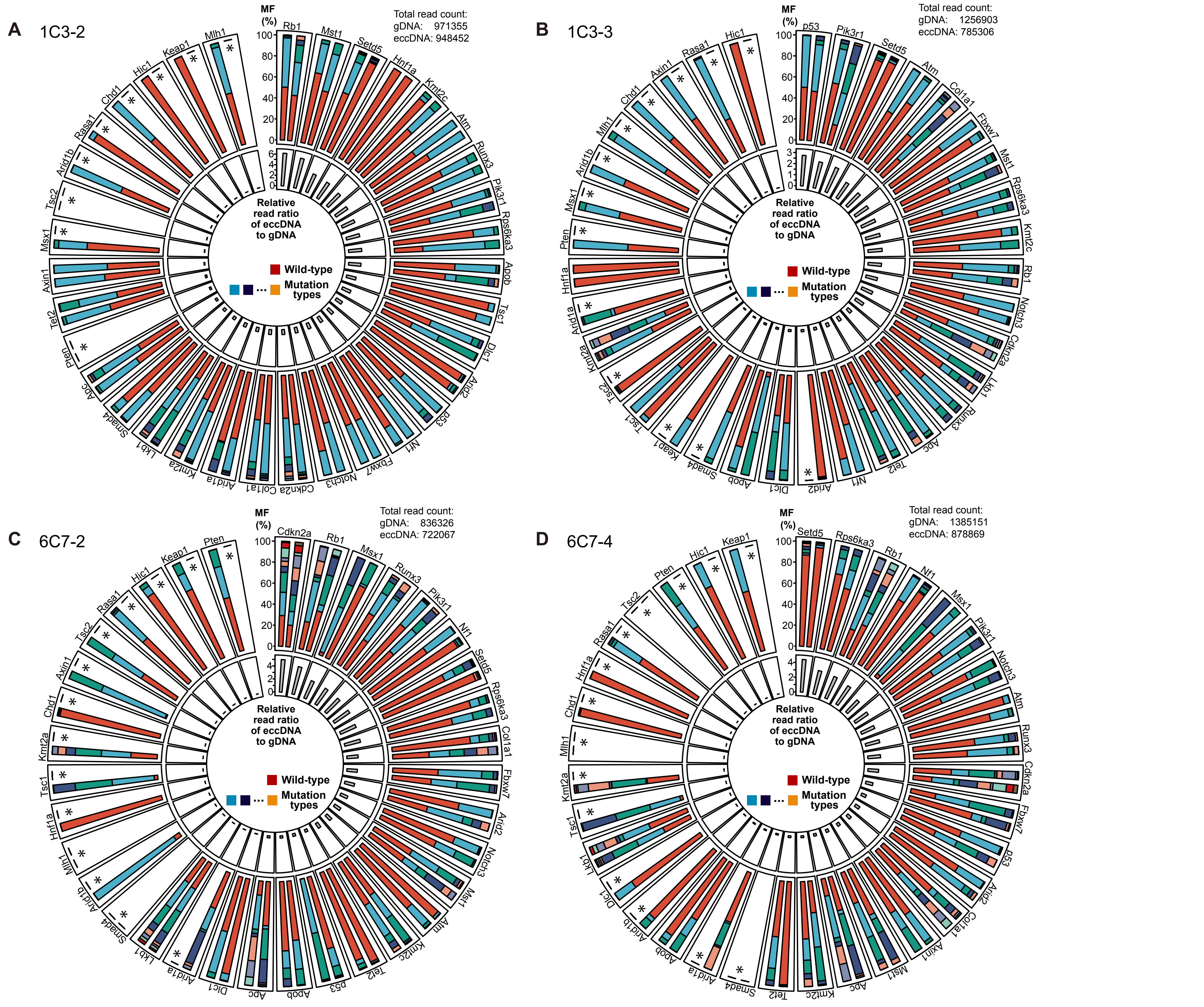
A 1C3-2
Total read count:
gDNA: 971355
eccDNA: 948452
B 1C3-3
Total read count:
gDNA: 1256903
eccDNA: 785306
C 6C7-2
Total read count:
gDNA: 836326
eccDNA: 722067
D 6C7-4
Total read count:
gDNA: 1385151
eccDNA: 878869
MF (%)
Relative read ratio of eccDNA to gDNA
Wild-type
Mutation types

Figure S12

A

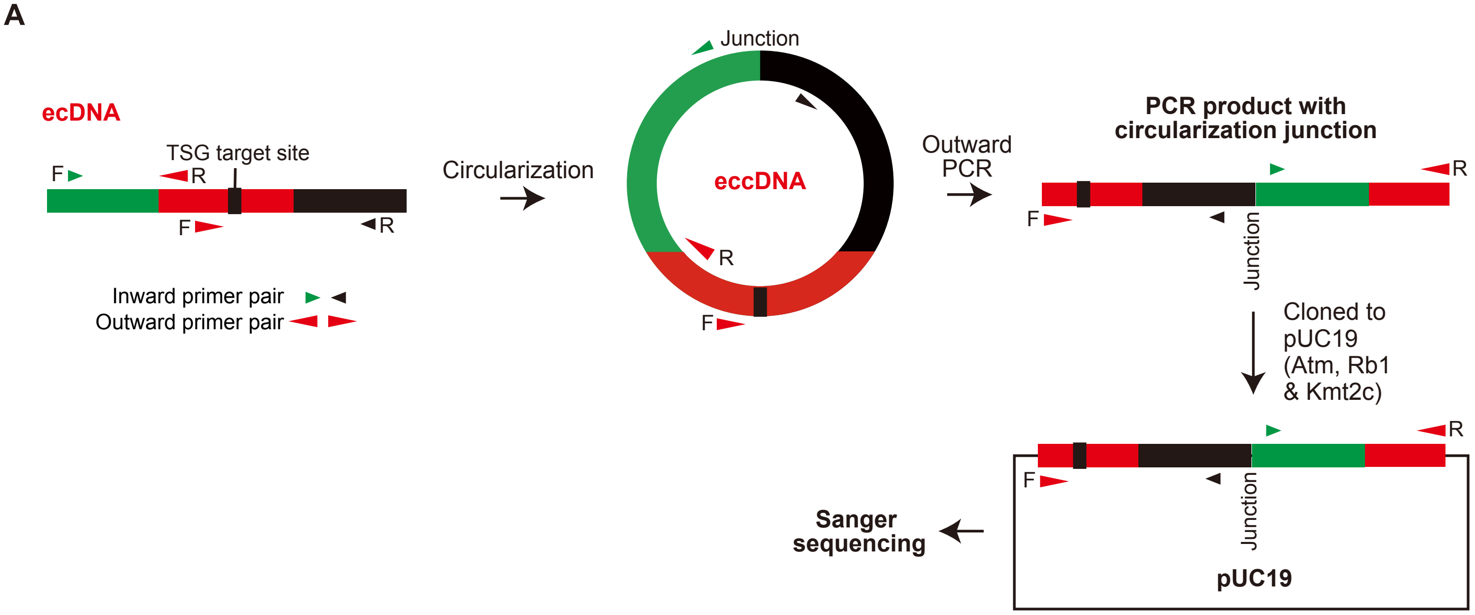

B

Target sequences for eccDNA generation

Note: Exon sequences in red; >< for circularization position and direction; Outward primer pairs for PCR underlined; CRISPR/Cas9 target sites indicated by PAM in bold.

Atm exon 25
ATGTTTATAATGTGTTTTCTATGTGCTCTATGCTATACCAGTTATTAAATACATTTTCCATTTTCTCATT
TGCTATCTT >TCTGTCATTCATTGGTGAAGAAATCAAAGTAGAAAAACATTGAGCGACTGCTCTAAAGTCC
TATTATTCCCATTTTCTTAAACATCATGTTTTTCCTTTCTGCAGGTTTTAGAGAAAGTCTCCGAATCGTT
TGG ATGTAGAAGTTTAGAAGACTTCATGATTTCTCACCTAGACTACCTGGTTTTGGAATGGCTGAACCTT
CAAGATACTG< AATATAGCTTATCTTCTTTTCCTTTTATGTTATTAAACTACACAAGCATTGAGGATTTCT
ATCGGTAGGCTGCAGCAGTGTTTCGACAACCCCCTCCTCCCTAGCTTCAAGTAGGAAGTAGCCTAGTTTT

Rb1 exon 8
ATTGTTTTTATTCTAATTTACCATTTT >TACAGAAACAGCTGCAATCCCCATTAATGGTTCACCTCGAAC
ACCCAGAAGAGGTCAGAACAGGAGCGCTCGGATAGCAAAACAACTAGAAAATGATACG**AGG** ATTATCGAGG
TTCTCTGTAAAGAACACGAGTGTAATATAGATGAGGTAATTTCTCTTACACTTTATTTAAAACAGCTGAA
GTAGATATATATGCTAAGTCTGCCTGTCA< ATATATACTTCTTGTGAGCATACTTTCTTTTTTTAAGTAGG

Kmt2c exon 36
TTCTCAGCCACCAGCAGTACCAAGGCCAAGGACTTCAGAGGGTTTTACTAGGCCCTCCAGTGCAAGACCA
GCC >CTCATGCCAAACCAGGATCCTTTTTTGCAAGCAGCACAAaaCCGAGTACCAGGTTTACCTGGCCCTT
TGATAAGGCCACCTGATACATGCTCCCAGACTCCCAGGCCACCTGGG**CCT** GGCCGTATAGACACATTCAC
TCATGCTTCCTCATCTGCTGTTCGTGATCCATATGATCAGCCTCCAGTGACTCCCAGGCCTCATTCTGAG
TCTTTCG< GAACTAGTCAAGTTGTTCACGATCTTGTTGACCGTCCAGTTCCTGGGTCAGAGGGAAACTTTA
